# Stellettin B Induces Cell Death in Bladder Cancer Via Activating the Autophagy/DAPK2/Apoptosis Signaling Cascade

**DOI:** 10.3390/md21020073

**Published:** 2023-01-21

**Authors:** Chun-Han Chang, Bo-Jyun Lin, Chun-Han Chen, Nham-Linh Nguyen, Tsung-Han Hsieh, Jui-Hsin Su, Mei-Chuan Chen

**Affiliations:** 1 School of Pharmacy, College of Pharmacy, Taipei Medical University, Taipei 110, Taiwan; 2 Department of Pharmacology, School of Medicine, College of Medicine, Taipei Medical University, Taipei 110, Taiwan; 3 Faculty of Chemical and Food Technology, HCMC University of Technology and Education, Ho Chi Minh 70000, Vietnam; 4 Joint Biobank, Office of Human Research, Taipei Medical University, Taipei 110, Taiwan; 5 Department of Science Education, National Museum of Marine Biology and Aquarium, Pingtung 944401, Taiwan; 6 Traditional Herbal Medicine Research, Center of Taipei Medical University Hospital, Taipei 110, Taiwan; 7 Ph.D. Program in Clinical Drug Development of Herbal Medicine, College of Pharmacy, Taipei Medical University, Taipei 110, Taiwan

**Keywords:** bladder cancer, stellettin B, apoptosis, autophagy, DAPK2

## Abstract

Bladder cancer (BC) is one of the most prevalent cancers worldwide. However, the recurrence rate and five-year survival rate have not been significantly improved in advanced BC, and new therapeutic strategies are urgently needed. The anticancer activity of stellettin B (SP-2), a triterpene isolated from the marine sponge *Rhabdastrella* sp., was evaluated with the MTT assay as well as PI and Annexin V/7-AAD staining. Detailed mechanisms were elucidated through an NGS analysis, protein arrays, and Western blotting. SP-2 suppressed the viability of BC cells without severe toxicity towards normal uroepithelial cells, and it increased apoptosis with the activation of caspase 3/8/9, PARP, and γH2AX. The phosphorylation of FGFR3 and its downstream targets were downregulated by SP-2. Meanwhile, it induced autophagy in BC cells as evidenced by LC3-II formation and p62 downregulation. The inhibition of autophagy using pharmacological inhibitors or through an ATG5-knockout protected RT-112 cells from SP-2-induced cell viability suppression and apoptosis. In addition, the upregulation of DAPK2 mRNA and protein expression also contributed to SP-2-induced cytotoxicity and apoptosis. In RT-112 cells, an FGFR3-TACC3-knockout caused the downregulation of DAPK2, autophagy, and apoptosis. In conclusion, this is the first study demonstrating that SP-2 exhibits potent anti-BC activity by suppressing the FGFR3-TACC3/Akt/mTOR pathway, which further activates a novel autophagy/DAPK2/apoptosis signaling cascade.

## 1. Introduction

As the 10th most common malignancy in the world, bladder cancer (BC) has been characterized by high recurrence rates, tumor heterogeneity, and resistance to chemotherapy, leading to a significant burden of cost on healthcare systems and compromised patient outcomes [1,2]. BC can be divided into non-muscle-invasive bladder cancer (NMIBC) and muscle-invasive bladder cancer (MIBC), two divergent molecular subtypes based on their histopathological profiles [3]. About 80% of BC patients are firstly diagnosed with NMIBC and with a 5-year survival rate of greater than 85%; however, the recurrence rate for NMIBC is 60–70%, and 30% of people experience disease progression to MIBC [4]. In addition to standard intravesical immunotherapy with Bacillus Calmette–Guérin (BCG) for intermediate- and high-risk NMIBC after the transurethral resection of the bladder (TURB) [5], approval for the immune checkpoint inhibitor (ICI) pembrolizumab was also provided for BCG-unresponsive NMIBC in 2020 [6]. The remaining 20% of patients present with MIBC and are diagnosed with a reported 5-year survival rate of 40–60%, and a drastic decrease occurs in the 5-year survival to 6% following disease progression to the metastatic stage [7,8]. Platinum-based chemotherapy has been the first choice for locally advanced and metastatic BC patients, but second-line therapeutic options are limited when patients encounter resistance or are ineligible for therapy [9,10]. However, ICIs have been approved as the second-line therapy for platinum-refractory patients or as the first-line therapy for patients who are cisplatin-ineligible and harbor tumors with a high PD-L1 level. Still, only 20% of patients can benefit from ICI treatments, and serious adverse events often lead to treatment discontinuation [11,12,13]. Hence, the development of novel therapeutic strategies remains an urgent need for patients with advanced BC.

Recent studies have focused on molecular subtyping to identify predictive biomarkers for successful treatment and to identify molecular targets for new drug discovery [14,15]. Gene mutations, copy-number variations, and rearrangements in the receptor tyrosine kinase-MAPK and PI3K-MTOR pathways are detected in ~ 70% of BC patients, which fosters the development of new therapies against these targets [16]. Currently, erdafitinib is the only fibroblast growth factor receptor (FGFR) inhibitor approved by the FDA for treating platinum-refractory metastatic urothelial carcinomas that harbor susceptible FGFR2/3 alterations [17]. Meanwhile, FGFR3 genetic alterations are frequently associated with the luminal papillary subtype in MIBC patients, and these tumors are reportedly lymphocyte-exclusionary and are less immunogenic [6,18]. Therefore, FGFR-targeting drugs may help in overcoming resistance to immunotherapy by releasing neoantigens and by priming the immune system [17].

Autophagy is a type of cell death triggered when cells face survival stress that promotes cell fitness, genome integrity, tissue homeostasis, and cell survival and growth [19]. When autophagy is activated, the ULK complex and VPS34 complex are activated to form a phagophore. Then, ATG4 cleaves LC3 to LC3-I, which is conjugated with phosphatidylethanolamine to form LC3-II by ATG3 and ATG7. The LC3-II and ATG5 complex contributes to phagophore elongation to form autophagosomes [19,20,21]. After that, the autophagosomes are conjugated with the lysosome to degrade the cellular components. Autophagy and apoptosis represent the two conserved intracellular processes that can regulate cell survival and death under stress conditions, and their crosstalk has also been reported [22]. For example, nonconjugated ATG5 is cleaved by calpain, and its product, ATG5tN, enters the mitochondria to promote the release of cytochrome c [23]. Caspase 8 can form a complex with death receptors, such as FAS-associated death domain (FADD), and can colocalize with ATG5, LC3, and p62 [24,25]. In this complex, the activation of caspase 8 occurs in an ATG5-, LC3-, and p62-dependent manner, demonstrating that autophagy can sometimes promote and induce apoptosis in cancer cells.

Marine natural products often provide a great variety of diverse structure types and promising biological activities, which may have a great potential as new drug leads. More than 20 candidates are in different stages of clinical trials [26,27]. Four of the nine FDA-approved marine drugs are derived from marine sponges [28]. For example, eribulin is a synthetic analogue of halichondrin B, which is a large polyether macrolide found in a variety of marine sponges [28,29]. Eribulin has been approved for patients with metastatic breast cancer who have previously received at least two chemotherapeutic regimens, including an anthracycline and a taxane regimen [30]. In addition, eribulin represents a better-manageable safety profile than taxane with a low incidence of peripheral neuropathy [31]. Antibody–drug conjugates (ADCs) are a group of immunological drugs designed for targeted therapy, where a cytotoxic agent is bound to a monoclonal antibody, targeting a tumor-specific antigen [32]. Enfortumab vedotin, an ADC consisting of a fully human monoclonal antibody specific for nectin-4 and monomethyl auristatin E (MMAE), was approved in 2019 for advanced BC patients who progressed on a regimen of platinum-based chemotherapy and PD1/PD-L1 inhibitors [33]. Auristatin is a synthetic analogue of dolastatin 10 (D10), which is one of the cytotoxic peptides from the Indian Ocean mollusk *Dolabella auricularia* [28]. Taken together, these findings indicate that developing new drugs from marine-derived natural products will provide a constructive solution for the treatment of advanced BC patients.

Stellettin B (SP-2) is a triterpene isolated from the marine sponge *Rhabdastrella* sp. which has been shown to induce autophagy and apoptosis in leukemia, NSCLC, and glioblastoma cells by interfering with the PI3K/Akt and mTOR signaling pathways [27,34,35]. However, SP-2 has not been tested in BC cells, and its detailed mode of action remains elusive. In this study, we investigated the anticancer activity and pharmacological mechanisms of SP-2 to reveal its potential for BC treatment.

## 2. Results

### 2.1. SP-2 Selectively Suppressed Cell Viability in BC Cells

**Figure 1:**
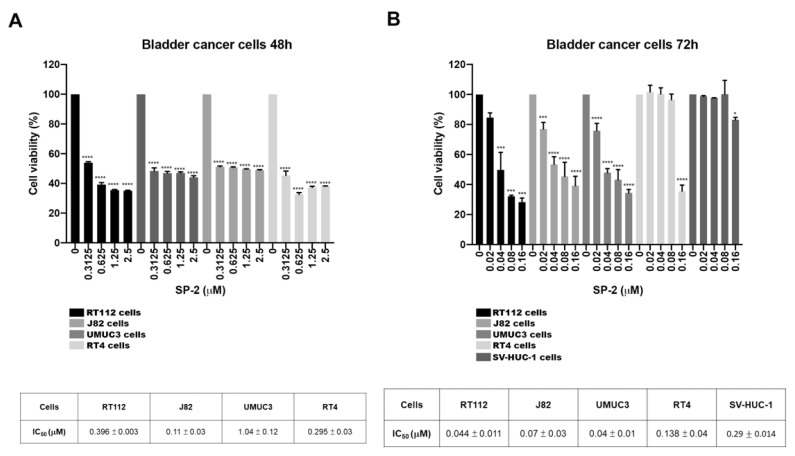
SP-2 selectively suppressed cell viability in bladder cancer (BC) cells. BC cells (RT-112, J82, UMUC3, and RT4) and normal uroepithelial cells (SV-HUC-1) were treated with different concentrations of SP-2 for 48 h (**A**) and 72 h (**B**), and cell viability was determined through MTT assay. * *p* < 0.05, *** *p* < 0.001, and **** *p* < 0.0001 compared with control group.

To determine the anticancer activity of stellettin B (SP-2) in bladder cancer (BC) cells, we performed MTT assays in several BC cell lines (RT-112, J82, UMUC3, and RT4). As shown in Figure 1A,B, the 72 h SP-2 treatment showed a more pronounced suppression of cell viability than the 48 h treatment without inducing severe cytotoxicity toward the normal uroepithelial SV-HUC-1 cells. Notably, the cell-killing effect of SP-2 at higher concentrations (0.08 μM and 0.16 μM) was more prominent in the RT-112 cells than in the other BC cell lines, meaning that RT-112 cells are an appropriate model for assessing the effects of SP-2 and its underlying molecular mechanism of action. Our findings showed that SP-2 exerted selective cytotoxicity against the BC cells.

### 2.2. SP-2 Induced Apoptosis in BC Cells

**Figure 2:**
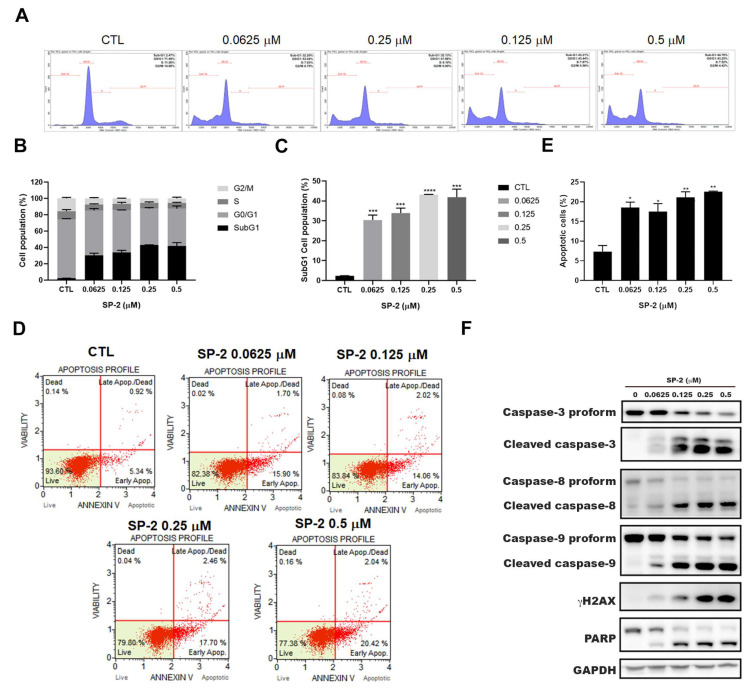
SP-2 induced accumulation of cells in the sub-G1 phase and apoptosis in RT-112 cells. (**A**–**C**) SP-2 induced sub-G1 cell population in RT-112 cells. Cells were treated with SP-2 at different concentrations for 48 h then were stained with PI, and cell cycle distribution was detected through flow cytometry (**A**). Quantitative data (**B**,**C**) are based on flow cytometry histograms and are presented as mean ± S.D. (**D**,**E**) SP-2 induced annexin-V-positive apoptotic cells in RT-112 cells. Quantitative data of apoptotic cells are presented as mean ± S.D. (**E**). Cells were treated with SP-2 at different concentrations for 48 h and stained with Annexin V/7-AAD. Apoptotic cells were detected through flow cytometry. (**F**) SP-2 increased levels of the cleaved forms of PARP; caspase 3, 8, and 9; and γH2AX in a concentration-dependent manner. Cells were treated with the indicated concentrations of SP-2 for 48 h, and cell lysates were immunoblotted using the indicated antibodies. * *p* < 0.05, ** *p* < 0.01, *** *p* < 0.001, and **** *p* < 0.0001 compared with control group.

To investigate the mechanism underlying the SP-2-induced repression of cell viability, we examined the effects of SP-2 on cell cycle progression. We found that the SP-2-treated cells stained with PI mainly accumulated in the sub-G1 phase (Figure 2A–C). We further used annexin V/7-AAD double staining to detect and quantify the percentage of the apoptotic population in the drug-treated cells. Consistent with this, SP-2 induced apoptosis in the RT-112 cells in a concentration-dependent manner (Figure 2D,E). Further, the dramatic induction of the sub-G1 phase and the increased apoptotic cells were in parallel with a significant activation of caspase 3, 8, and 9; γH2AX; and poly-(ADP-ribose) polymerase (PARP) (Figure 2F). SP-2-induced apoptosis was also observed in the RT4 cells (Figure S1A). Meanwhile, the cell membranes started blebbing after 24 h of treatment, and apoptotic body-like morphological changes were also observed after 48 h of treatment in the RT-112 cells (Figure S2). Taken together, these results indicated that SP-2 significantly induced apoptosis in the BC cells.

### 2.3. The Effects of SP-2 on the Phosphorylation of Receptor Tyrosine Kinases (RTKs) and Protein Kinases in RT-112 Cells

**Figure 3:**
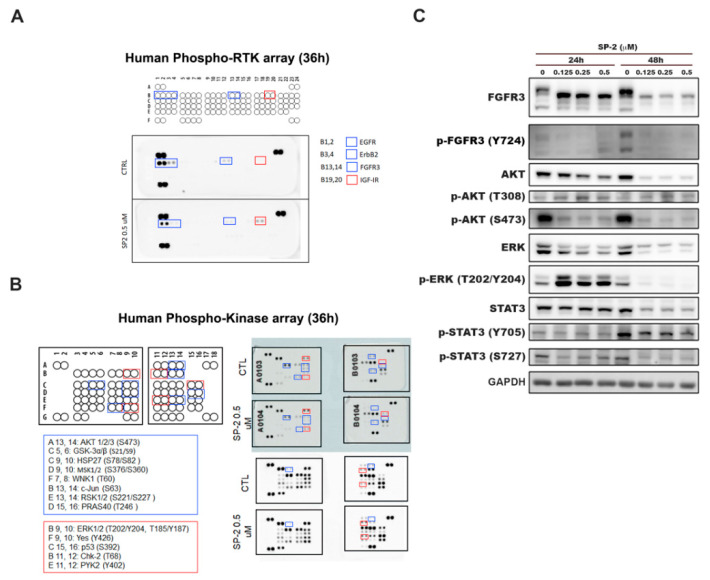
The effects of SP-2 on the phosphorylation profiles in RT-112 cells. (**A**,**B**) SP-2 affected the phosphorylation of various receptor tyrosine kinases (RTKs) (**A**) and protein kinases (**B**) in RT-112 cells. Cells were treated with SP-2 (0.5 μM) for 36 h, and cell lysates were applied to phosphoprotein array analysis. Protein dots in the blue box indicate increased phosphorylation, and protein dots in the red box indicate decreased phosphorylation after SP-2 treatment. (**C**) SP-2 significantly downregulated p-FGFR3 and its downstream signaling pathways. Cells were treated with different concentrations of SP-2 for the indicated times, and cell lysates were immunoblotted using the indicated antibodies.

To comprehensively understand the pharmacological mechanisms of SP-2, we employed protein kinase arrays to evaluate the changes in the phosphorylation of RTKs and protein kinases. The data suggested that SP-2 decreased the levels of p-FGFR3, p-EGFR, and p-ErbB2 in the RT-112 cells (Figure 3A). Next, we further examined the effects of SP-2 on several important downstream survival signals using a protein kinase array. The results showed that the phosphorylation of Akt, GSK3α/β, and several kinases was downregulated by SP-2 after 36 h of treatment (Figure 3B). The aberrant activation of FGFR3 signaling has a well-established role in the development of BC [14]. Thus, FGFR3 has been proposed as a therapeutic target, and a number of inhibitors are currently in clinical trials [36]. Since RT-112 cells harbor an FGFR3-TACC3 gene fusion, which results in the constitutive activation of FGFR3 signaling [37], we further confirmed the expression level of FGFR3 in the SP-2-treated cells with a Western blot analysis. The results showed that SP-2 caused a band shift of the FGFR3-TACC3 fusion protein to a lower molecular weight and that its phosphorylation was dramatically downregulated (Figure 3C). Importantly, the phosphorylation levels of the downstream targets of FGFR3, such as p-AKT and p-STAT3, were significantly reduced by SP-2 (Figure 3C). The effect of SP-2 on the band shift of the FGFR3-TACC3 fusion protein was also observed in the RT4 cells (Figure S1A). These findings indicated that SP-2 has the potential to inhibit the FGFR3 signaling pathway in BC cells.

### 2.4. SP-2 Activated Autophagy in BC Cells

**Figure 4:**
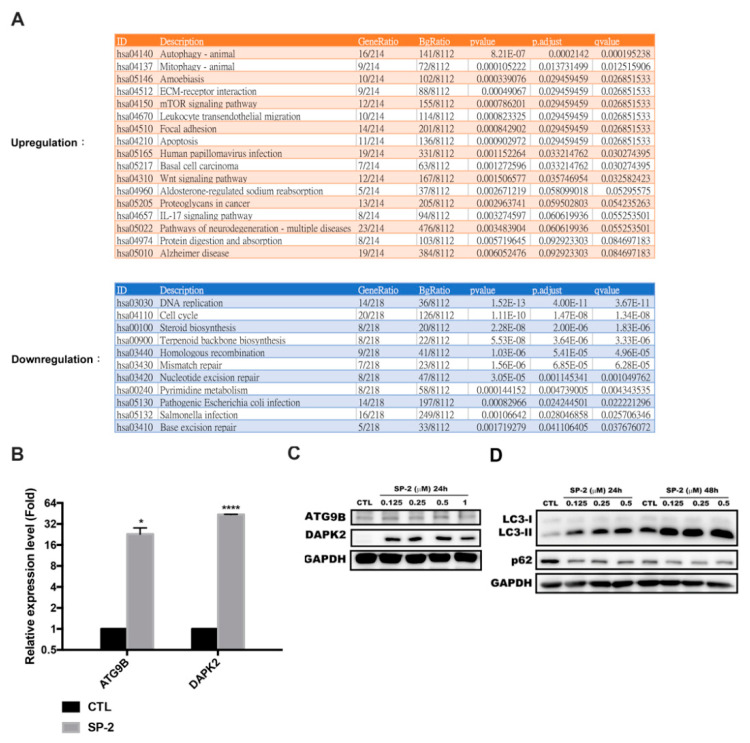
SP-2 induced autophagy in RT-112 cells. (**A**) SP-2 upregulated autophagy and downregulated steroid biosynthesis according to NGS-based pathway analysis. Cells were treated with 0.5 μM SP-2 for 24 h. The data were analyzed through NGS as described in Materials and Methods. (**B**,**C**) Expression levels of mRNAs (**B**) and proteins (**C**) of ATG9B and DAPK2 with SP-2 treatment. Cells were treated with various concentrations of SP-2 for 24 h, and mRNA levels and proteins were analyzed through RT-qPCR and Western blotting. (**D**) Concentration-dependent effect of SP-2 on the conversion of endogenous LC3-I to LC3-II. Cells were treated with different concentrations of SP-2 for 24 h and 48 h, and cell lysates were immunoblotted using the indicated antibodies. * *p* < 0.05 and **** *p* < 0.0001 compared with the control (CTL) group.

To further investigate gene and signaling pathway regulation in response to SP-2, Next-generation sequencing (NGS) was performed to characterize differentially expressed genes upon SP-2 treatment. The functional enrichment analysis of the Kyoto Encyclopedia of Genes and Genomes (KEGG) pathways among gene clusters was also performed to identify any affected signaling pathways. We found that the autophagy pathway was upregulated in the SP-2-treated cells (Figure 4A, upper table). Meanwhile, the DNA replication, cell cycle, and steroid biosynthesis pathways were downregulated with SP-2 treatment (Figure 4A, lower table). We further validated the NGS data by performing an RT-qPCR and Western blotting to analyze some differentially expressed genes in the SP-2-treated RT-112 cells. Autophagy-related protein 9B (ATG9B) and death-associated protein kinase 2 (DAPK2) have been reported as a regulator of autophagy and a modulator of mTORC1 activity and the autophagy level, respectively [38,39]. We confirmed that SP-2 treatment induced the mRNA and the protein expression of ATG9B and DAPK2 (Figure 4B,C). Significant LC3-II formation and P62 degradation were also detected in the SP-2-treated RT-112 cells in a concentration-dependent manner (Figure 4D). DAPK2 induction and autophagy were also detected in the RT4 cells treated with SP-2 (Figure S1B). These data indicated that SP-2 activated the autophagy pathway in the BC cells.

### 2.5. SP-2-Induced Apoptosis Was Dependent on Autophagy in RT-112 Cells

**Figure 5:**
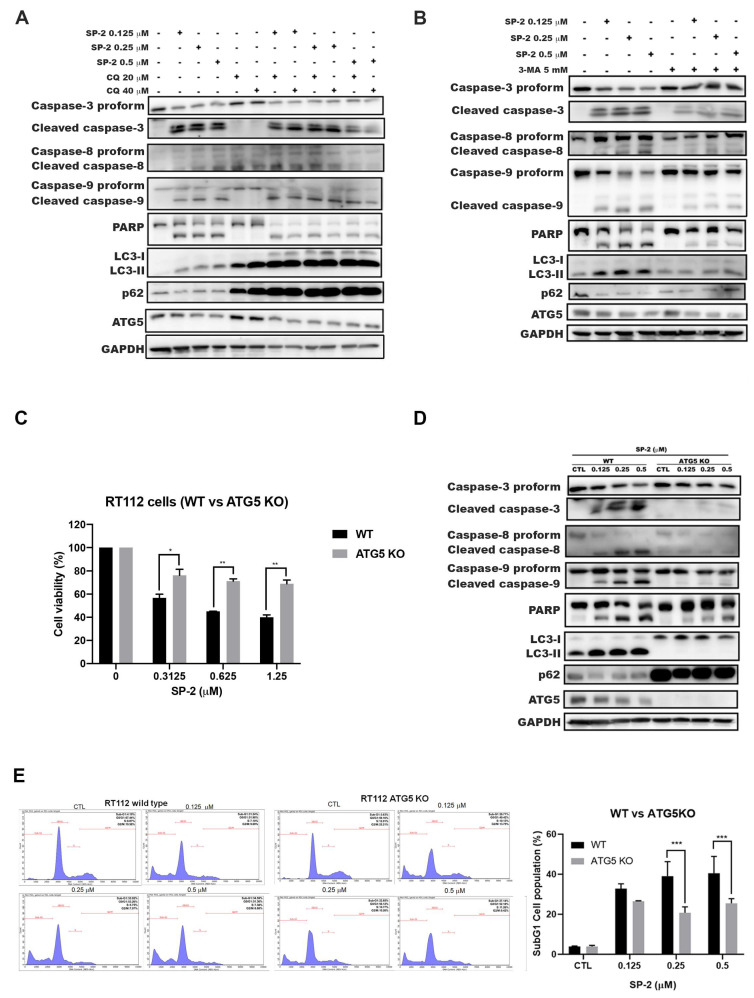
Knockout of ATG5 reduced SP-2-induced sub-G1 accumulation and apoptosis in RT-112 cells. (**A**,**B**) Inhibition of autophagy with either CQ (**A**) or 3-MA (**B**) reduced SP-2-induced apoptosis in RT-112 cells. The cells were treated with SP-2 for 48 h in the presence or absence of an autophagy inhibitor (CQ or 3-MA), and cell lysates were analyzed through Western blotting. (**C**–**E**) ATG5-knockout reduced SP-2-induced cytotoxicity (**C**), apoptosis (**D**), and accumulation in the sub-G1 phase (**E**) in RT-112 cells. Cells were treated with indicated concentrations of SP-2 for 48 h, and cell viability (**C**) and cell cycle distribution (**E**) were determined with MTT assay and through flow cytometry, respectively. * *p* < 0.05, ** *p* < 0.01, and *** *p* < 0.001. Cell lysates from 48 h SP-2 treatment were analyzed through Western blotting (**D**).

To further investigate the role of autophagy in SP-2-induced cytotoxicity, we first used a pharmacological approach by blocking autophagy with chloroquine (CQ) and 3-methyladenine (3-MA). The CQ-induced inhibition of the autophagosome and lysosome fusion resulted in increased levels of LC3-II and p62, whereas the 3-MA-induced inhibition of autophagy was mediated by the inhibition of LC3-II formation (Figure 5A,B). Combination with either the CQ or 3-MA autophagy inhibitors attenuated the SP-2-induced activation of caspase 3, 8, and 9 as well as that of PARP (Figure 5A,B). These findings suggested that targeting the modulation of autophagy played an important role in SP-2-induced cell death in the RT-112 cells. We further inhibited autophagy in the RT-112 cells by knocking out the *ATG5* gene [40]. As shown in Figure 5C–E, the ATG5-knockout rescued the SP-2-induced suppression of cell viability (Figure 5C), reversed the SP-2-induced accumulation of γH2AX and the activation of apoptosis (Figure 5D), and led to cell cycle accumulation in the sub-G1 phase (Figure 5E). These results indicated that the activation of autophagy was required for SP-2-induced apoptosis in the RT-112 cells.

### 2.6. DAPK2-Knockdown Ameliorated SP-2-Induced Apoptosis in RT-112 Cells

**Figure 6:**
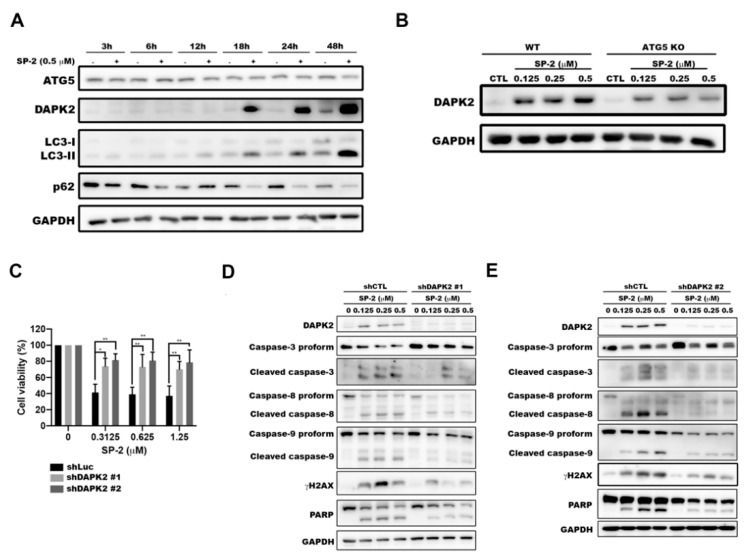
DAPK2 potentiated SP-2-induced apoptosis in RT-112 cells. (**A**) SP-2 induced autophagy and the DAPK2 level in a time-dependent manner. (**B**) ATG5-knockout reduced the upregulation of DAPK2 in RT-112 cells. Cells were treated with different concentrations of SP-2 for 48 h, and cell lysates were immunoblotted using the indicated antibodies. (**C**–**E**) DAPK2-knockdown protected SP-2-induced cytotoxicity (**C**) and apoptosis (**D**,**E**) in RT-112 cells. Cells were treated with different concentrations of SP-2 for 48 h, and cell viability and apoptosis were determined with MTT assay and through Western blotting, respectively. * *p* < 0.05 and ** *p* < 0.01.

Previous reports revealed that DAPK2 is a novel regulator of autophagy through its direct association with mTORC1 to suppress mTOR activity to activate autophagy [39]. Our results revealed the upregulation of DAPK2 after 18 h of treatment in parallel with the significant induction of LC3-II (Figure 6A). The ATG5-knockout significantly reduced SP-2-induced DAPK2 expression (Figure 6B), suggesting that DAPK2 is downstream of the autophagy pathway. Next, we knocked down DAPK2 to further investigate the role of DAPK2 in SP-2-induced apoptosis. The DAPK2-knockdown significantly protected cells from SP-2-induced cytotoxicity (Figure 6C) and apoptosis (Figure 6D,E). Taken together, our results suggested that DAPK2 may play an important role in switching the cells from excessive autophagy to apoptosis in SP-2-induced cell death in the RT-112 cells.

### 2.7. FGFR3-TACC3 Fusion Was Required for SP-2-Induced Autophagy in RT-112 Cells

**Figure 7:**
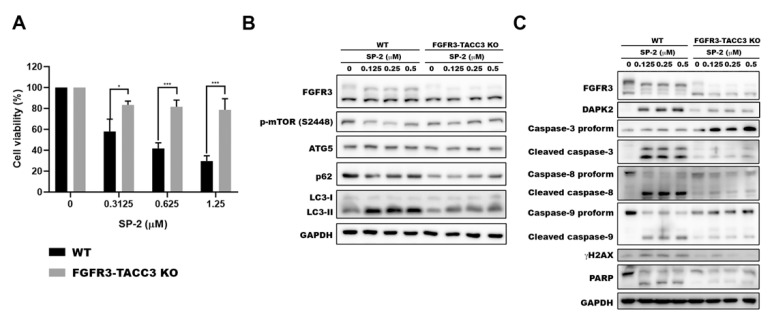
FGFR3-TACC3 fusion was essential to SP-2-induced autophagy and apoptosis in RT-112 cells. (**A**) FGFR3-TACC3 knockout significantly reversed SP-2-reduced cell viability. Cells were treated with different concentrations of SP-2 for 48 h, and cell viability was determined with MTT assay. * *p* < 0.05 and *** *p* < 0.001. (**B**) FGFR3-TACC3 knockout decreased SP-2-indcued autophagy. Cells were treated with different concentrations of SP-2 for 48 h, and cell lysates were immunoblotted using the indicated antibodies. (**C**) FGFR3-TACC3 knockout protected RT-112 cells from SP-2-induced apoptosis. Cells were treated with different concentrations of SP-2 for 48 h, and cell lysates were immunoblotted using the indicated antibodies.

FGFR3 is frequently activated through mutation or overexpression and has become an actionable therapeutic target in BC [18]. Specifically, an FGFR3-transforming acid coiled-coil 3 (TACC3) fusion is another genetic aberration resulting in the constitutive activation of downstream signaling in BC [41]. In addition to SP-2-triggered autophagy-dependent apoptosis (Figure 5), we observed that SP-2 inhibited the activity of FGFR3-TACC3 in the RT-112 cells as evidenced by a decrease in p-FGFR3 and its downstream signaling pathways (p-AKT and p-STAT3) (Figure 3C). Therefore, we hypothesized that SP-2-mediated autophagy may result from the inhibition of the FGFR3-TACC3/AKT/mTOR axis. Therefore, we generated the FGFR3-TACC3-specific knockout (KO) clone using the CRISPR/Cas9 technique. The results showed that the FGFR3-TACC3 knockout dramatically rescued SP-2-induced cytotoxicity in the RT-112 cells (Figure 7A). In the wild-type cells, SP-2 decreased mTOR phosphorylation and upregulated autophagy in a concentration-dependent manner; however, this phenomenon was reversed in the FGFR3-TACC3-KO cells (Figure 7B). Surprisingly, the FGFR3-TACC3-KO could also rescue cells from SP-2-mediated DAPK2 induction and apoptotic cell death (Figure 7C). The results suggested that SP-2 exerted its anticancer activity by abrogating the FGFR3-TACC3/pAKT/mTOR signaling pathway, which subsequently activated autophagy and apoptosis in the RT-112 cells.

## 3. Discussion

Platinum-based chemotherapy has been the standard of care for locally advanced and metastatic BC patients, but second-line therapeutic options are limited when patients encounter resistance or are ineligible for therapy [9,10]. Marine natural products offer a great variety of diverse structure types and promising biological activities, which may have a great potential as new drug leads [26,27]. Isomalabaricane triterpenoid SP-2 was first isolated by separating the diazomethane-treated dichloromethane extract from the sponge *Jaspis stellifera* collected on the reef flats of Fiji in 1981 [42]. SP-2 can be also collected from Somalian waters (*Stelletta* sp.) [43]; Cape Wilberforce, Australia (*Stelletta* sp.) [44]; Mindanao, Philippines (*R. globostellata*) [45]; Hainan Island (*R*. aff. *distincta*) [46]; and Xisha Island, South China Sea (*Geodia Japonica*) [47,48]. The SP-2 in our study was collected from the marine sponge *Rhabdastrella* sp. by hand via SCUBA off the coast of Penghu, Taiwan. Previous studies have demonstrated that SP-2 shows promising anticancer activity in human promyelocytic leukemia HL60 cells [49], human chronic myeloid leukemia [38], glioblastoma [34], non-small cell lung carcinoma [50], hepatocellular carcinoma [51], and oral squamous cell carcinoma [52]. Despite the promising anticancer activity of SP-2 in various human cancer types, the isomalabaricane scaffold remains largely unexplored as a potential anticancer lead and presents challenges for drug discovery because of a lack of material. However, a recent study has shown the total synthesis of several stellettins within the family of isomalabaricane triterpenoids [53], suggesting it is possible to continue to inspire more total synthesis work on isomalabaricanes to provide sufficient quantities of promising candidates for biological investigations. In our study, the results showed that SP-2 suppressed BC cell viability without displaying severe toxicity toward the normal uroepithelial cells, SV-HUC-1 cells (Figure 1B). SP-2 induced apoptosis in the RT-112 and RT4 cells as evidenced by an increase in the population of cells in the sub-G1 phase and by the activation of caspase 3, 8, and 9; γH2AX; and PARP (Figure 2C,F and Figure S1A). However, a low concentration of SP-2 (0.1 nM) showed neuroprotective effects against cell apoptosis and oxidative stress through PI3K/Akt, the MAPK pathway, and Nrf2/HO-1 upregulation in the SH-SY5Y cells [54]. SP2 treatment alone (0.1 nM) did not alter the expression level of p-Akt or p-ERK. Notably, pretreatment with SP2 reversed the 6-OHDA-induced downregulation of p-Akt and p-ERK [54]. Our results showed that SP-2 induced p-ERK after 24 h of treatment and significantly reduced p-ERK expression after 48 h of treatment (Figure 3C). These findings suggested that SP-2 may exhibit drug-concentration-related or context-dependent mechanisms of action and effects. SP-2 also inhibited angiogenesis by reducing HIF-1α and VEGF in the glioblastoma cells [35], suggesting that SP-2 has the potential to regulate the HIF-1α-associated pathways and to change cellular and genomic regulation [55,56,57]. Recently, Peng et al. found that SP-2 can sensitize glioblastoma cells to temozolomide chemotherapy by suppressing the PI3K-mediated homologous recombination repair [58]. The study indicated that SP-2 represents a promising drug candidate for drug combination.

SP-2 has been shown to induce autophagic cell death in oral cancer cells. The combination of the autophagy inhibitor 3-MA attenuates SP-2-suppressed cell viability [52]. Our NGS analysis identified that the genes related to the autophagy pathway, such as ATG9B and DAPK2, were upregulated after treatment with SP-2 (Figure 4B,C). A previous study revealed that DAPK2 associates with the components of mTORC1 and regulates mTORC1 activity and the autophagy levels [39]. The pharmacological inhibitors of autophagy and the ATG5-knockout rescued the cells from apoptosis, indicating that the activation of autophagy was required for SP-2-induced apoptosis in the RT-112 cells (Figure 5). Meanwhile, the ATG5-knockout also reversed SP-2-induced DAPK2 expression, and the DAPK2-knockdown rescued the cells from SP-2-mediated apoptosis (Figure 6B). It has been reported that DAPK2 exhibits proapoptotic functions in cancer cells. The activation of E2F1 in osteosarcoma cells increased endogenous DAPK2 in parallel with cell death. KLF6 expression in H1299 cells increased DAPK2 levels, which was also accompanied by cell death [59]. In the future, transcriptional regulators need to be identified to activate DAPK2 expression with SP-2 in RT-112 cells.

The aberrant activation of FGFR3 signaling is frequently observed in patients with BC, and the corresponding targeted therapy has gradually emerged as an alternative treatment in advanced BC patients [14]. In 2019, the US FDA approved the first target therapy erdafitinib, which targets the FGFR3 and FGFR2 mutation genes in advanced BC patients [60]. Specifically, RT-112 and RT4 cells harbor an FGFR3-TACC3 gene fusion that causes the constitutive activation of FGFR3 signaling [37]. The results showed that SP-2 treatment caused a band shift of FGFR3-TACC3 to a lower molecular weight, and its phosphorylation was dramatically suppressed in the RT-112 and RT4 cells (Figure 3C and Figure S1A). Therefore, it was worth elucidating the mechanism of FGFR3-TACC3 band shifting with SP-2 treatment. Importantly, the phosphorylation of the downstream targets, such as p-AKT and p-STAT3, was significantly reduced by SP-2 in the RT-112 cells (Figure 3C). Knocking out FGFR3-TACC3 diminished the effect of SP-2 on viability, autophagy, and apoptosis in the RT-112 cells (Figure 7). Our findings demonstrated that SP-2 was able to target the upstream factor FGFR3-TACC3 gene fusion to activate its anticancer activity in the BC cells. These results suggested that SP-2 is a promising agent for the treatment of BC patients with an FGFR3–TACC3 fusion. Meanwhile, FGFR3 genetic alterations are frequently associated with the luminal papillary subtype in MIBC patients, and these tumors are reportedly lymphocyte-exclusionary and are less immunogenic [6,18]. Santiago-Walker et al. evaluated anti-PD-L1 therapy outcomes in advanced BC patients with and without FGFR alternations, and the results revealed that the median overall survival in FGFR-altered patients was lower than that in FGFR-wildtype patients [61]. Therefore, SP-2 may help to overcome resistance to immunotherapy by releasing neoantigens and by priming the immune system in the future.

In our study, we observed that DAPK2 was upregulated after treatment with SP-2 and that the DAPK2-knockdown could rescue apoptosis in the RT-112 cells. However, there were some limitations to our study that need to be clarified in the future. First, the role of DAPK2 in the activation of apoptosis was ambiguous, and the detailed mechanism of DAPK2 in triggering apoptosis remains elusive. In 1999, DAPK2 was identified and characterized as a calcium/calmodulin (Ca^2+^/CaM)-dependent protein kinase [62]. The study found that the activation or overexpression of DAPK2 is related to the activation of apoptosis. Changing the lysine residue at amino acid site 52 to alanine resulted in a decrease in apoptosis, suggesting that the expression of DAPK2 plays a proapoptotic role. However, another study found that knocking down DAPK2 in A549 and U2OS cells can lead to the activation of apoptosis through treatment with the TNF-related apoptosis-inducing ligand (TRAIL), including caspase 3/8/9 and PARP [63]. These results suggested that the absence of DAPK2 can resensitize U2OS and A549 cells for treatment with TRAIL in cancer therapy. It was noted that the basal expression levels of DAPK2 in the U2OS and A549 cells were higher than those in the RT-112 cells and that the induction of DAPK2 was stronger after treatment with SP-2. This may be one of the reasons for the difference in the results of our study and those of other studies on DAPK2’s role in the activation of apoptosis. In addition, DAPK2 reportedly contributes to the induction of autophagy by phosphorylating Beclin-1 on Thr119, the core autophagic machinery protein, causing it to dissociate from its inhibitor, Bcl-XL [64]. Therefore, it is worthy to examine whether SP-2-induced DAPK2 also contributes to the activation of autophagy, which may form a positive feedback loop between autophagy and DAPK2.

## 4. Materials and Methods

### 4.1. Cell Culture and Reagents

RT-112, UMUC-3, J82, and SV-HUC-1 cells were purchased from American Type Culture Collection (Manassas, VA, USA). RT4 cells were purchased from Bioresource Collection and Research Center (Hsinchu, Taiwan). RT-112-ATG5-KO cells were provided by Dr. Chun-Han Chen (Department of Pharmacology, College of Medicine, Taipei Medical University, Taiwan) [40]. RT-112 and RT-112-ATG5-KO cells were grown in RPMI 1640, RT4 cells were grown in McCoy’s 5A, UMUC-3 and J82 cells were grown in EMEM, and SV-HUC-1 cells were grown in F12K. All the cells were cultured at 37 °C in the presence of 5% CO_2_ supplemented with 10% FBS and 1 × antibiotic–antimycotic. The materials for cell cultures were purchased from Thermo Fisher Scientific (Waltham, MA, USA). Puromycin was purchased from InvivoGen (San Diego, CA, USA). Chloroquine diphosphate salt was purchased from Sigma (St. Louis, MO, USA). 3-MA was purchased from Cayman (Ann Arbor, MI, USA). Anti-mouse and anti-rabbit IgGs were purchased from Jackson ImmunoResearch Laboratories (West Grove, PA, USA).

### 4.2. Generation of FGFR3 Exon 18::TACC3 Exon 11 Fusion Gene Knockout in RT-112 Cells

The FGFR3-TACC3 fusion gene knockout RT-112 cells were generated using CRISPR/Cas9 technology. One sgRNA, targeting the region in front of the 13th coding exon of NM_000142 (chr4:1739992), and the other one, targeting the region after the 12th coding exon of NM_006342 (chr4:1740002), were separately cloned into lenti-sgRNA-hygro plasmid from Addgene (Addgene plasmid # 104991; Cambridge, MA, USA). The following targeting sites were used: 5′-TACAGGGGCCCTGGGGACAC-3′ for FGFR3-sg and 5′-GGTGAGTGCCCGGGCCACCG-3′ for TACC3-sg. These two sgRNA plasmids, used for putative deletion on only the FGFR3-TACC3 fusion gene locus, were cotransfected with lenti-Cas9-Blast (Addgene plasmid # 52962) into RT-112 cells using Lonza^®^ 4D Nucleofector™ X Unit (Lonza, Basel, Switzerland). At two days posttransfection, transfected cells were selected using 200 µg/mL of hygromycin (InvivoGen, San Diego, CA, USA) and 100 µg/mL of blasticidin (InvivoGen, San Diego, CA, USA) for one week. Viable cells were diluted into a 96-well plate for the isolation of single-cell clones. The FGFR3-TACC3 fusion gene knockout cells were confirmed through Western blot analysis and DNA sequencing of the genomic regions.

### 4.3. Isolation and Identification of Stellettin B

Stellettin B (SP-2), isolated from the marine sponge *Rhabdastrella* sp., was provided by Dr. Jui-Hsin Su (National Museum of Marine Biology and Aquarium, Taiwan). Specimens of the marine sponge *Rhabdastrella* sp. were collected by hand via SCUBA off the coast of Penghu, Taiwan, at a depth of 10 to 15 m and were stored in a freezer until extraction. The sponge *Rhabdastrella* (Thiele, 1903) sp. belongs to the class Demospongiae, order Tetractinellida (Marshall, 1876), and family Ancorinidae (Schmidt, 1870) [65]. The frozen bodies of marine sponge *Rhabdastrella* sp. (1.2 kg, wet wt) were freeze dried; following this, the resulting dry sponge (250 g) was extracted exhaustively with MeOH (1L × 5). Then, the MeOH extract was subjected to further partitioning between H_2_O and CH_2_Cl_2_. The CH_2_Cl_2_ layer (10.5 g) was subjected to silica gel column chromatography and was eluted with n-hexane in EtOAc (0–100%, gradient) to yield ten fractions. Fraction 8 (1250 mg) was fractioned with LH-20 and with pure acetone to afford 5 subfractions (8-L1−8-L5). Subfractions 8-L4 and 8-L5 were combined (750 mg) and separated with normal-phase HPLC (n-hexane:acetone ratio of 3:1) to afford stellettin B (200 mg). The structure of stellettin B was identified through NMR spectroscopic analyses (Figures S3 and S4), and a stock solution (10 mM) was prepared by dissolving stellettin B in DMSO and was stored at −20 °C.

### 4.4. Cell Viability Assay

Cells were seeded into 96-well plates and were cultured overnight. Cells were then treated with various concentrations of compounds for 48 or 72 h for the cell viability MTT (3-(4,5-Dimethylthiazol-2-yl)-2,5 diphenyltetrazolium bromide) assay. Cell viability was determined by the absorbance of the treatment group versus that of the DMSO-treated control group (which means the cell viability of the control group was 100%). The IC_50_ value was determined by 50% cell viability after the treatment of drugs for 48 or 72 h compared with control.

### 4.5. Flow Cytometry Assay

Cells were seeded in 6-well plates and were treated with DMSO or various concentration compounds for 48 h. After the treatment, cells were trypsinized and washed with 1 mL PBS. The samples were fixed at −20 °C with 75% ethanol and at −20 °C overnight and were stained with propidium iodide (80 ug/mL), which contains 0.1% Triton-X 100 and 100 μg/mL of RNaseA. For the analysis of apoptosis, cells were collected and stained with the Muse^®^ Annexin V & Dead Cell Kit (Luminex, Austin, TX, USA) for 20 min. Finally, the samples were analyzed with the Guava^®^ Muse^®^ Cell Analyzer (Luminex, Austin, TX, USA).

### 4.6. Western Blot and Lentivirus System

Cells were seeded into 6-well plates overnight and treated with the indicated compounds at various concentrations for the indicated times. Proteins were extracted using a lysis buffer from whole-cell lysates, were fractionated in SDS-PAGE gels, and were transferred onto polyvinylidene difluoride (PVDF) membrane for protein blot analysis.

The primary antibodies for detection of the indicated protein were obtained from the following sources: caspase 3, LC3, and GAPDH, which were purchased from Novus (Littleton, CO, USA), as well as caspase 8, caspase 9, PARP, γH2AX, AKT, p-AKT (T308), p-AKT (S473), STAT3, p-STAT3 (Y705), and ATG5, which were purchased from Cell signaling (Dallas, TX, USA). P62 was purchased from GeneTex (Irvine, CA, USA). FGFR3 and p-FGFR3 were purchased from Abcam (Cambridge, UK). DAPK2 was purchased from Arigo Biolaboratories (Hsinchu, Taiwan). DAPK2 protein expression was stably knocked down using shRNA expressing lentiviral particles against DAPK2 (#1, TRCN0000199968; #2, TRCN0000001718), and shRNA-targeting luciferase was used as a negative control (TRCN0000072249); both were purchased from National RNAi Core Facility (Academia Sinica, Taipei, Taiwan). The cells were seeded into a 6-well plate for transduction. Stable knockdown cells were selected and maintained with puromycin (2 μg/mL).

### 4.7. Phosphoprotein Array Analysis

Cells were seeded into 10 cm dishes and were exposed to SP-2 (0.5 μM) for 36 h. Cell pellets were collected through scrapping, were washed by PBS, and were subjected to protein array analysis according to the manufacture’s instruction. The Proteome Profiler™ Human Phospho-RTK Array Kit was used to determine the relative phosphorylation of human receptor tyrosine kinases (R&D Systems, Cat. No. ARY001B, Minneapolis, MN, USA), and the Proteome Profiler™ Human Phospho-Kinase Array Kit was used to determine the relative levels of human protein kinase phosphorylation (R&D Systems, Cat. No. ARY003C, Minneapolis, MN, USA).

### 4.8. Next-Generation Sequencing (NGS)

Cells were seeded into 6 cm dishes and treated with SP-2 (0.5 μM) for 24 h. After the treatment, cells were scraped, washed with PBS, and lysed with TRIzol reagent (Thermo Fisher Scientific, Waltham, MA, USA) to isolate RNA. For library preparation and sequencing, the purified RNA was used to prepare the sequencing library with the TruSeq Stranded mRNA Library Prep Kit (Illumina, San Diego, CA, USA) according to the manufacturer’s recommendations. After the generation of double-strand cDNA and adenylation on 3′ ends of DNA fragments, the adaptors were ligated and purified with the AMPure XP system (Beckman Coulter, Beverly, MA, USA). The quality of the libraries was assessed on the Agilent Bioanalyzer 2100 system and on a Real-Time PCR system. The qualified libraries were then sequenced on an Illumina NovaSeq 6000 platform with 150 bp paired-end reads generated by Genomics BioSci & Tech Co. (New Taipei City, Taiwan).

### 4.9. Bioinformatics Analysis

The bases of a low quality and sequences from adapters in raw data were removed using the program fastp (version 0.20.0) [66]. The filtered reads were aligned to the reference genomes using HISAT2 (version 2.1.0) [67]. The Subread package of the software FeatureCounts (v2.0.1) was applied for the quantification of gene abundance [68]. Differentially expressed genes were identified using DESeq2 (version 1.28.0) [69]. The functional enrichment analysis of Gene Ontology terms and Kyoto Encyclopedia of Genes and Genomes (KEGG) pathways among gene clusters was implemented in an R package called clusterProfiler (version 4.0.0) [70,71,72].

### 4.10. Reverse Transcription Quantitative PCR

Cells were seeded into a 6-well plate and treated with SP-2 for 24 h. The total RNA of treated cells was isolated using the TRIzol reagent (Thermo Fisher Scientific, Waltham, MA, USA) and was reverse transcribed into complementary DNA by using a HiScript I TM First Strand cDNA Synthesis Kit (Bionovas, Toronto, ON, Canada). Quantitative PCR was performed in duplicate with gene-specific primers using a RealQ Plus 2x Master Mix Green Kit (Ampliqon, Odense, Denmark). For ATG9B, the primers were forward 5′-CCCCTCATACAAGAAGCTCCC-3′ and reverse 5′-TGCAGGTTGAGCCTGTGTTG-3′; for DAPK2, the primers were forward 5′-CATCCTTGAGCTAGTGTCTGGA-3′ and reverse 5′-GGATCTGCTTAATGAAGCTGGT-3′.

### 4.11. Statistical Analysis

Data are presented as the mean ± SD. Multiple *t*-test and one-way ANOVA were performed to obtain statistical significance. The data were considered significant when the *p*-value was lower than 0.05 (* *p* < 0.05, ** *p* < 0.01, *** *p* < 0.001, and **** *p* < 0.0001).

## 5. Conclusions

In conclusion, we identified that SP-2, a natural product isolated from the marine sponge, exhibited promising anti-bladder-cancer activity. SP-2 suppressed the FGFR3-TACC3/Akt/mTOR pathway, which resulted in the activation of autophagy and subsequently activated apoptosis in a DAPK2-dependent manner.

## Data Availability

The data presented in this study are available on request from the corresponding author.

## Supplementary Materials

The following supporting information can be downloaded at https://www.mdpi.com/article/10.3390/md21020073/s1, Figure S1. SP-2 induced autophagy and apoptosis in RT4 cells; Figure S2. Morphological changes of RT-112 cells after treatment with SP-2, Figure S3. ^1^H NMR spectrum of SP-2 in CDCl_3_ at 400 MHz; and Figure S4. ^13^C NMR spectrum of SP-2 in CDCl_3_ at 100 MHz.

## Abbreviations

3-methyladenine	(3-MA)
Antibody–drug conjugates	(ADCs)
Autophagy-related protein 9B	(ATG9B)
Bacillus Calmette–Guérin	(BCG)
Bladder cancer	(BC)
Chloroquine	(CQ)
Death-associated protein kinase 2	(DAPK2)
Dolastatin 10	(D10)
FAS-associated death domain	(FADD)
Fibroblast growth factor receptor	(FGFR)
Immune checkpoint inhibitor	(ICI)
Kyoto Encyclopedia of Genes and Genomes	(KEGG)
Muscle-invasive bladder cancer	(MIBC)
Knockout	(KO)
Monomethyl auristatin E	(MMAE)
Next-Generation Sequencing	(NGS)
Non-muscle-invasive bladder cancer	(NMIBC)
Poly-(ADP-ribose) polymerase	(PARP)
Receptor tyrosine kinases	(RTKs)
