# Supplementary material for: Stellettin B Induces Cell Death in Bladder Cancer Via Activating the Autophagy/DAPK2/Apoptosis Signaling Cascade
Source: Mar Drugs. 2023 Jan 21;21(2):73. doi: 10.3390/md21020073 (PMC9966069; doi:10.3390/md21020073)

Figure 2F

Caspase 3 proform

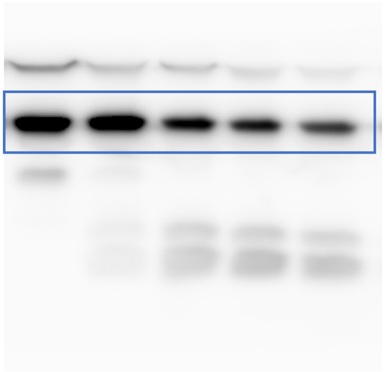

Cleaved caspase 3

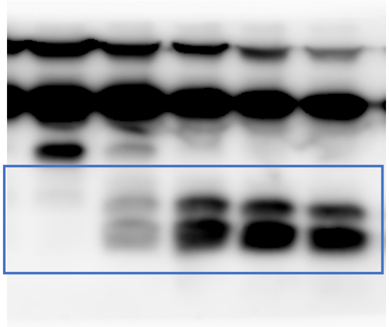

Caspase 8

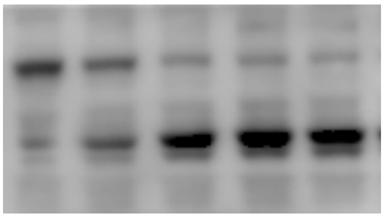

Caspase 9

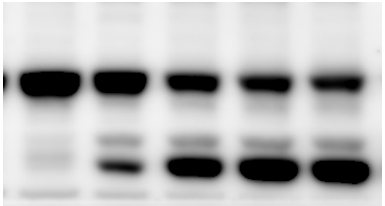

γH2AX

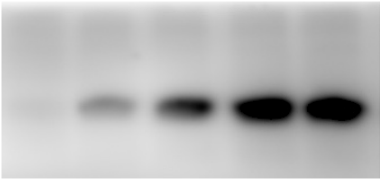

PARP

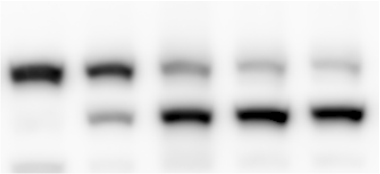

GAPDH

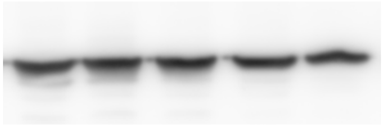

Figure 3C

FGFR3

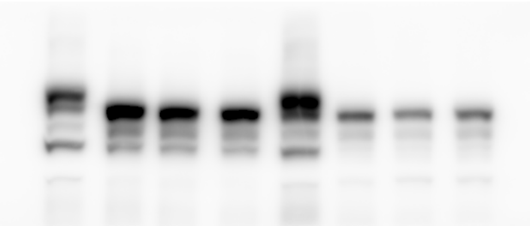

p-FGFR3 (Y742)

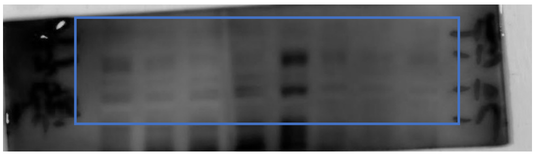

AKT

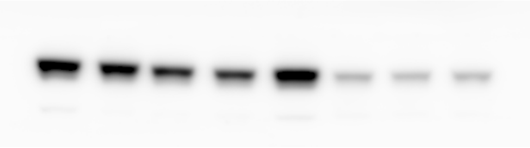

p-AKT (T308)

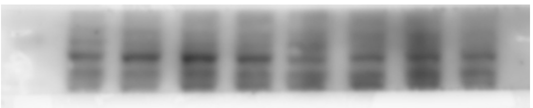

pAKT (S473)

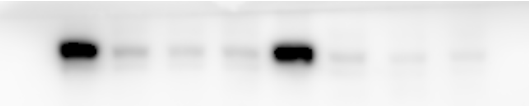

ERK

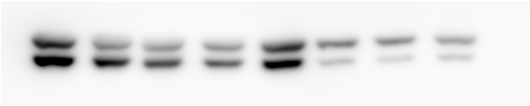

p-ERK (T202/Y204)

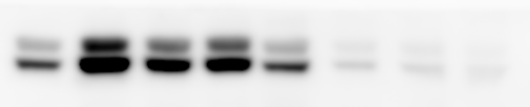

STAT3

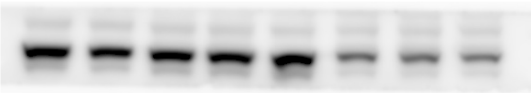

p-STAT3 (Y705)

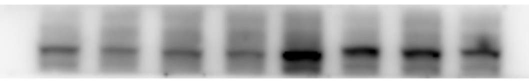

p-STAT3 (S727)

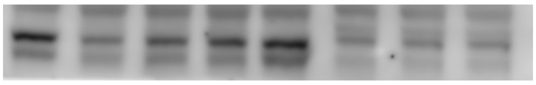

GAPDH

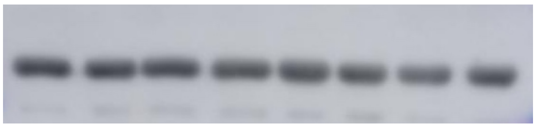

Figure 4C

ATG9B

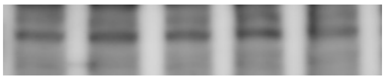

DAPK2

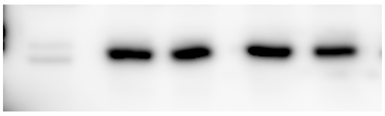

GAPDH

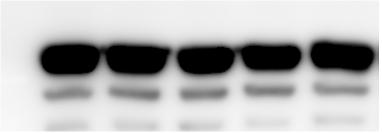

Figure 4D

LC3

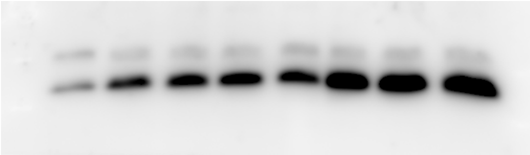

p62

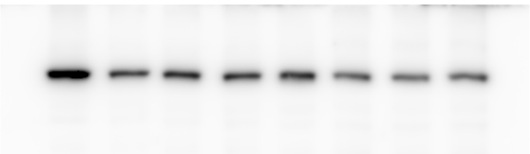

GAPDH

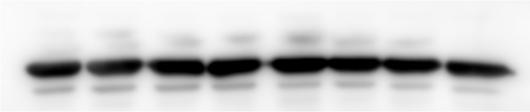

Figure 5A

C3 proform

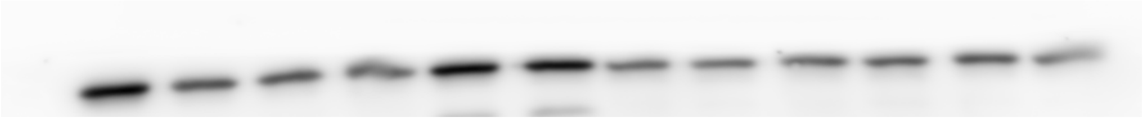

Cleaved caspase 3

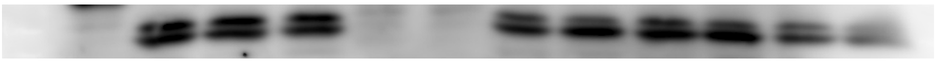

Caspase 8

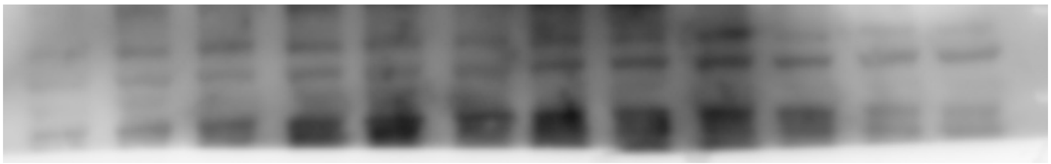

Caspase 9

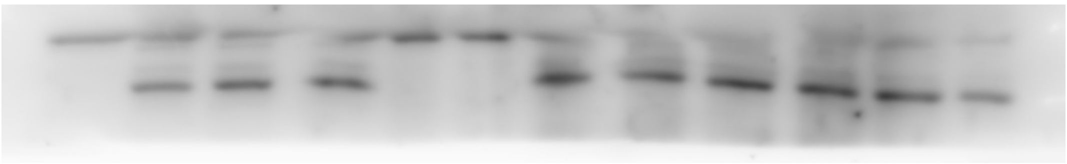

PARP

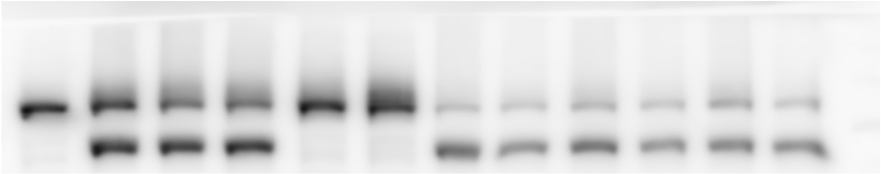

LC3

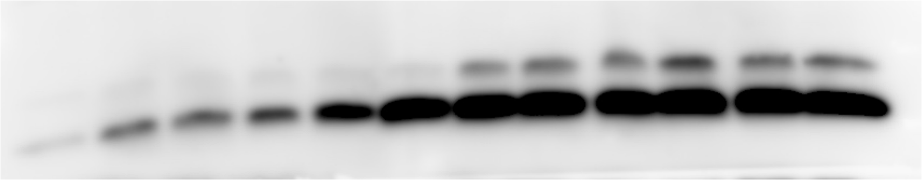

p62

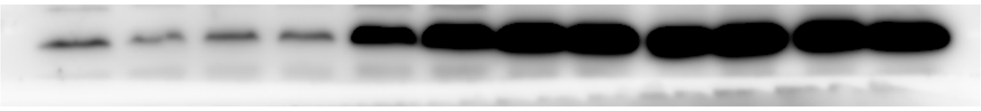

ATG5

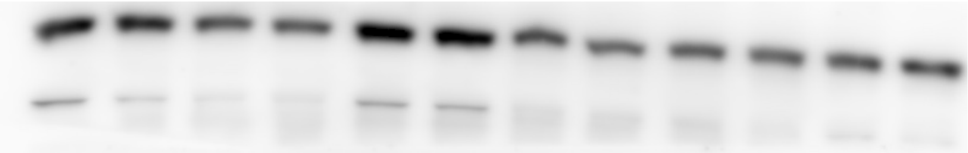

GAPDH

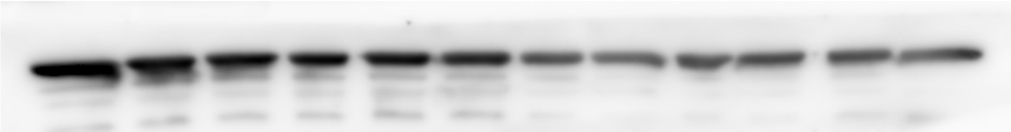

Figure 5B

Caspase 3 proform

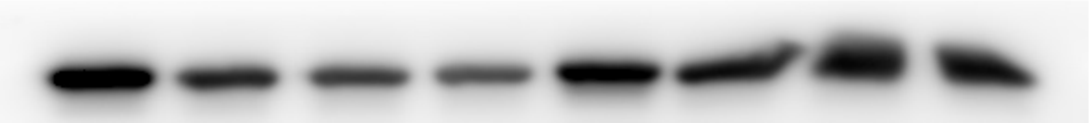

Cleaved Caspase 3

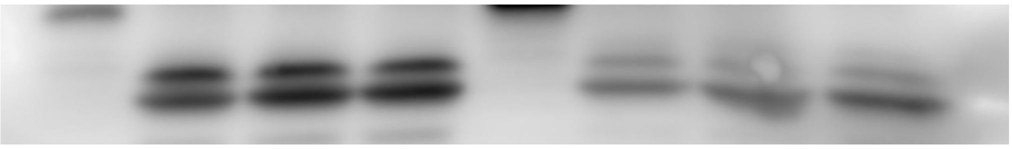

Caspase 8

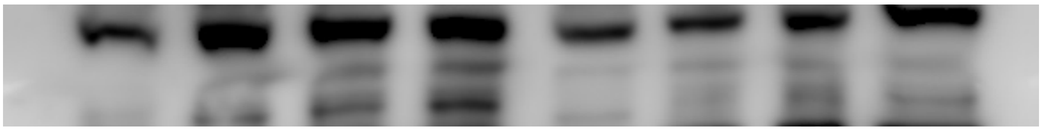

Caspase 9

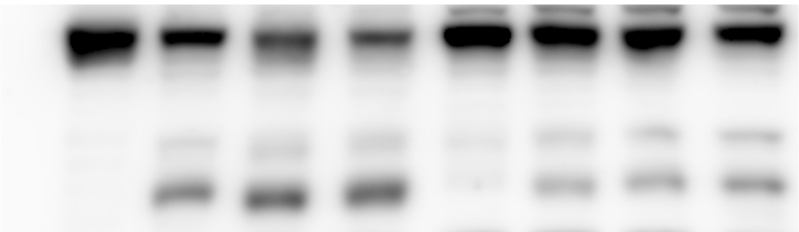

PARP

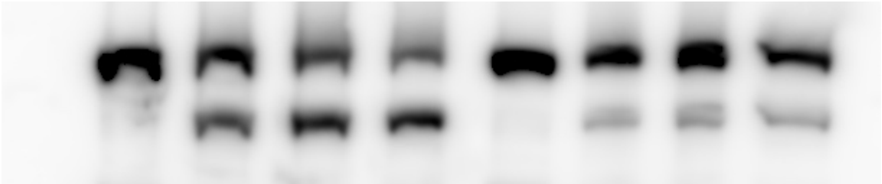

LC3

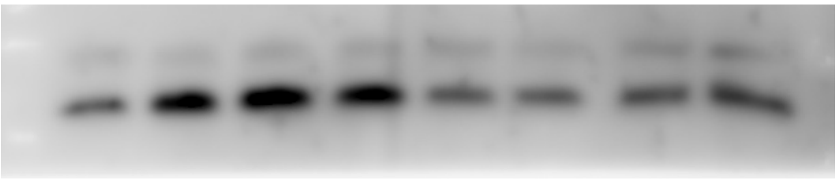

p62

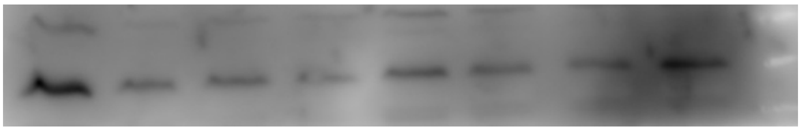

ATG5

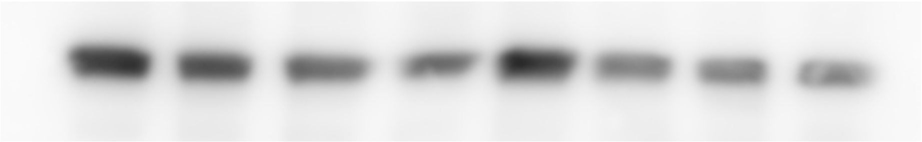

GAPDH

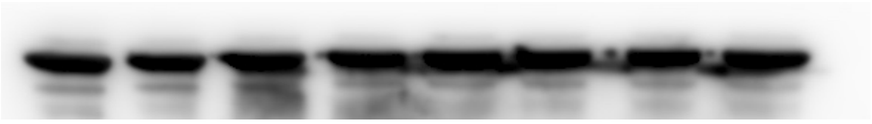

Figure 5D

Caspase 3 pro form

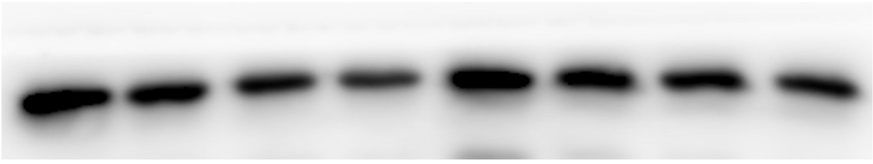

Cleaved Caspase 3

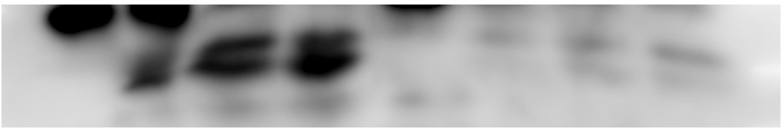

Caspase 8

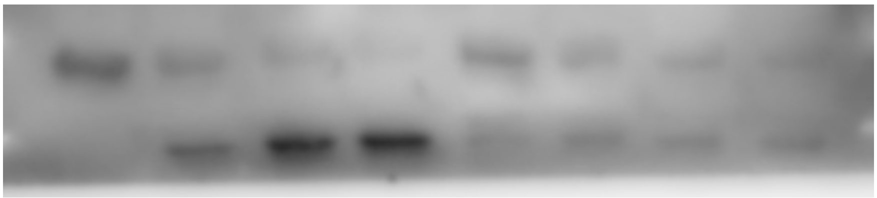

Caspase 9

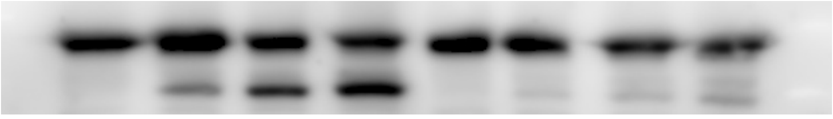

PARP

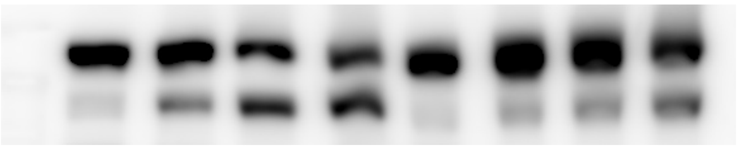

LC3

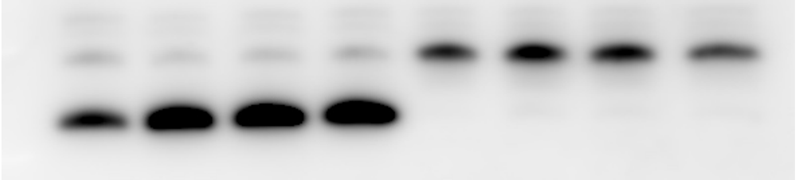

p62

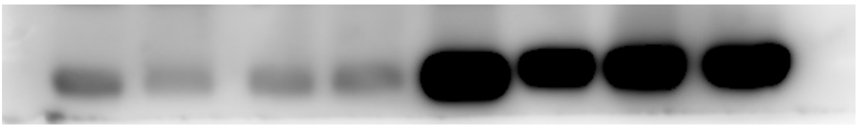

ATG5

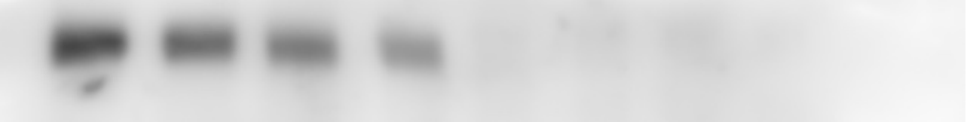

GAPDH

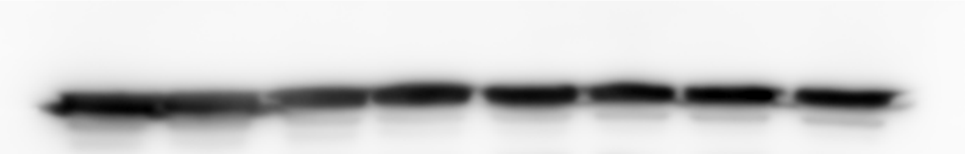

Figure 6A

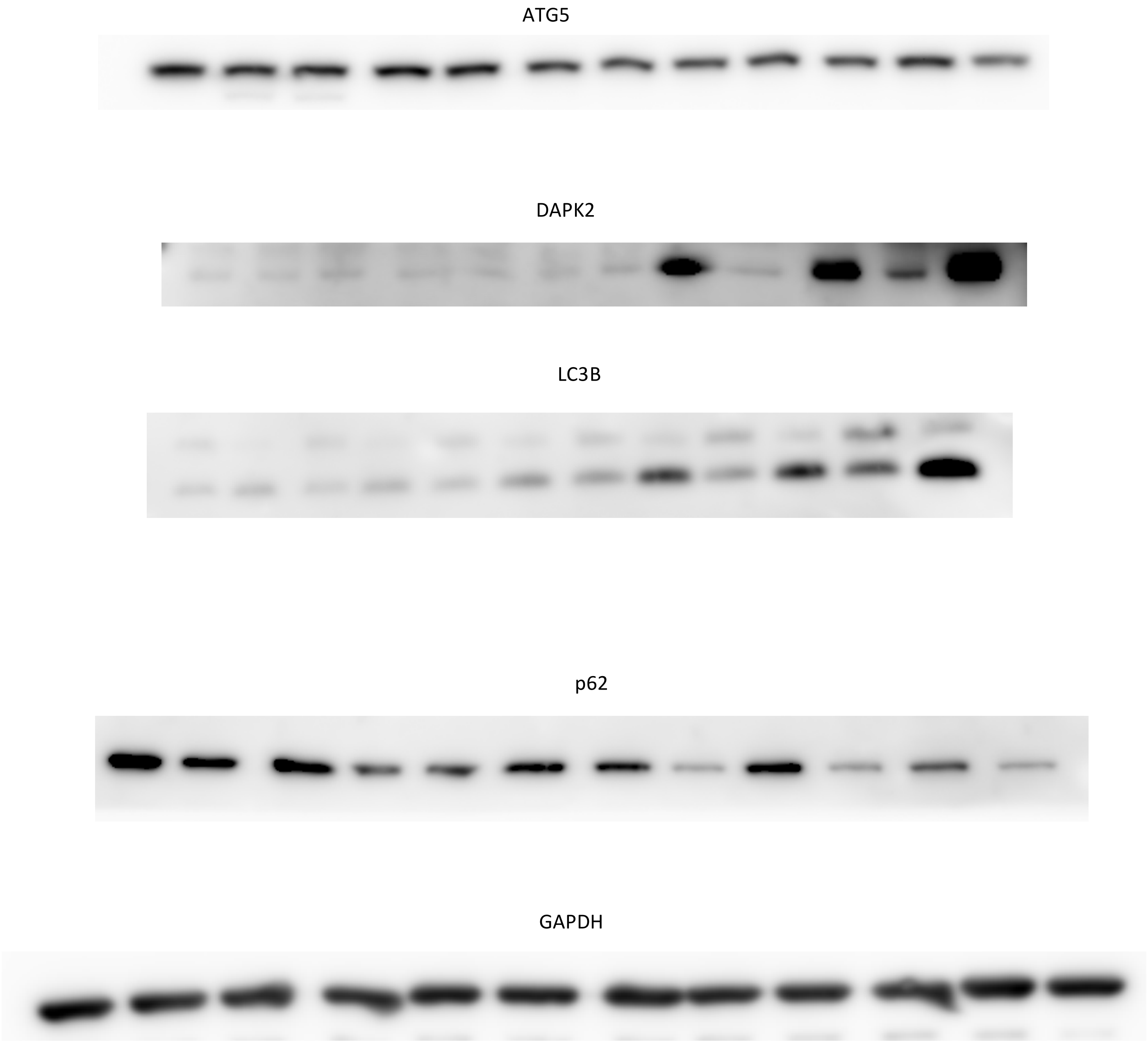

Figure 6B

DAPK2

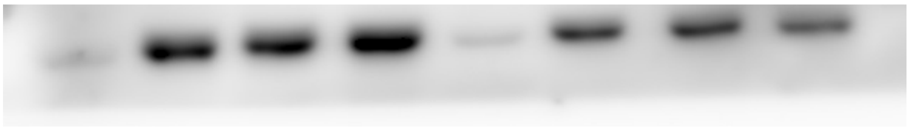

GAPDH

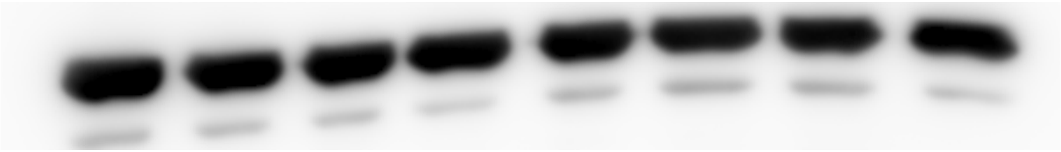

Figure 6D

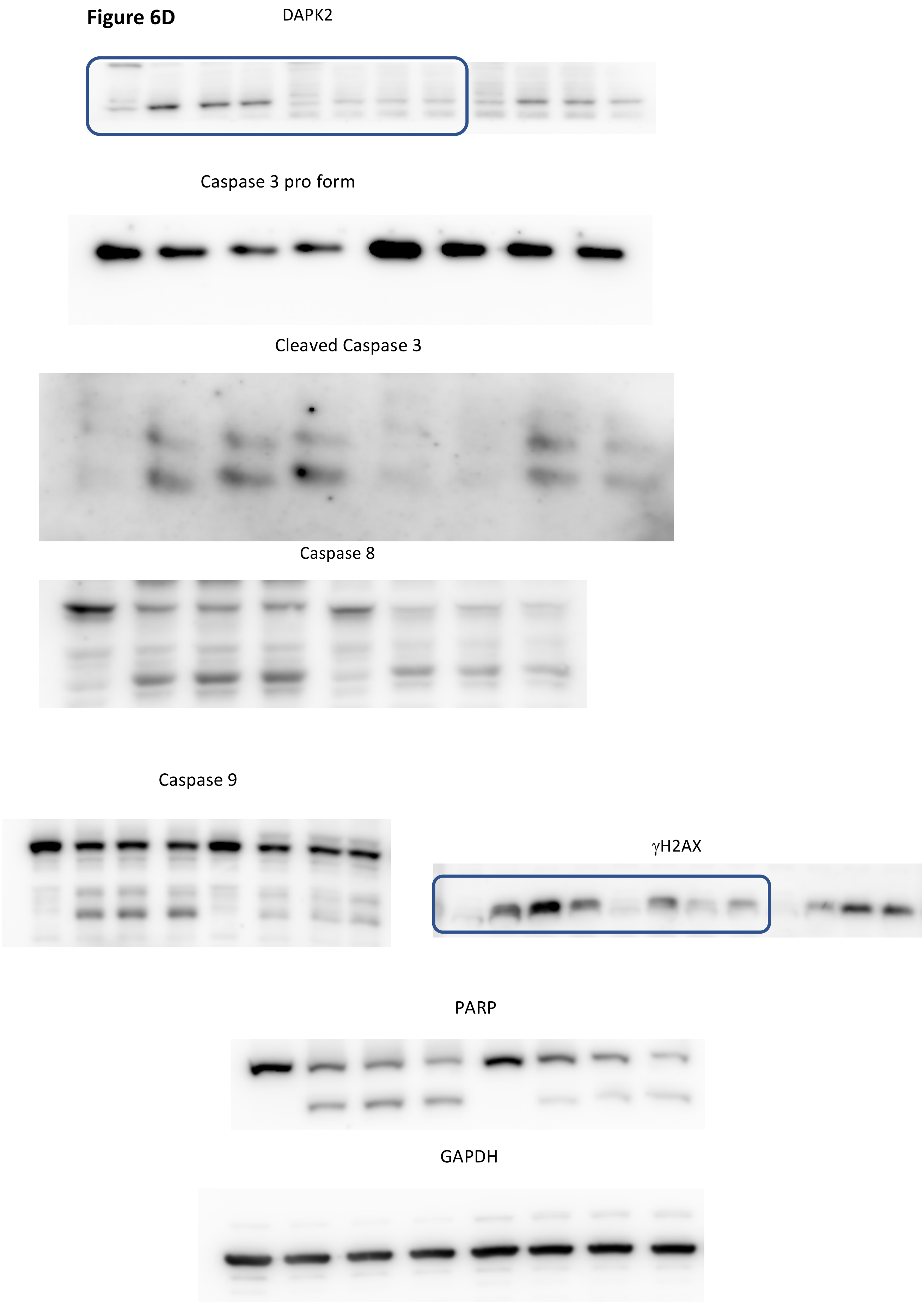
DAPK2
Caspase 3 pro form
Cleaved Caspase 3
Caspase 8
Caspase 9
γH2AX
PARP
GAPDH

Figure 6E

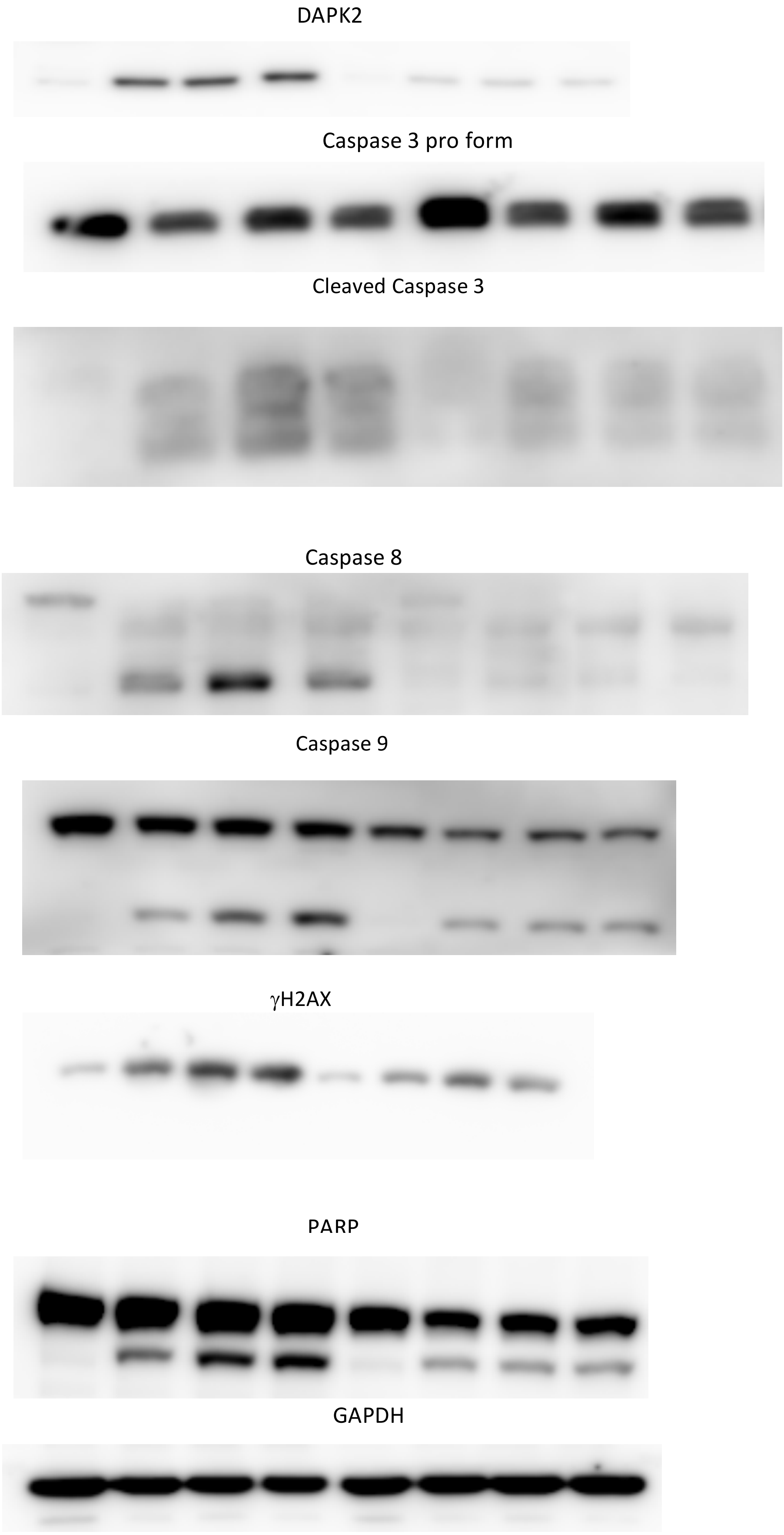
DAPK2
Caspase 3 pro form
Cleaved Caspase 3
Caspase 8
Caspase 9
γH2AX
PARP
GAPDH

Figure 7B

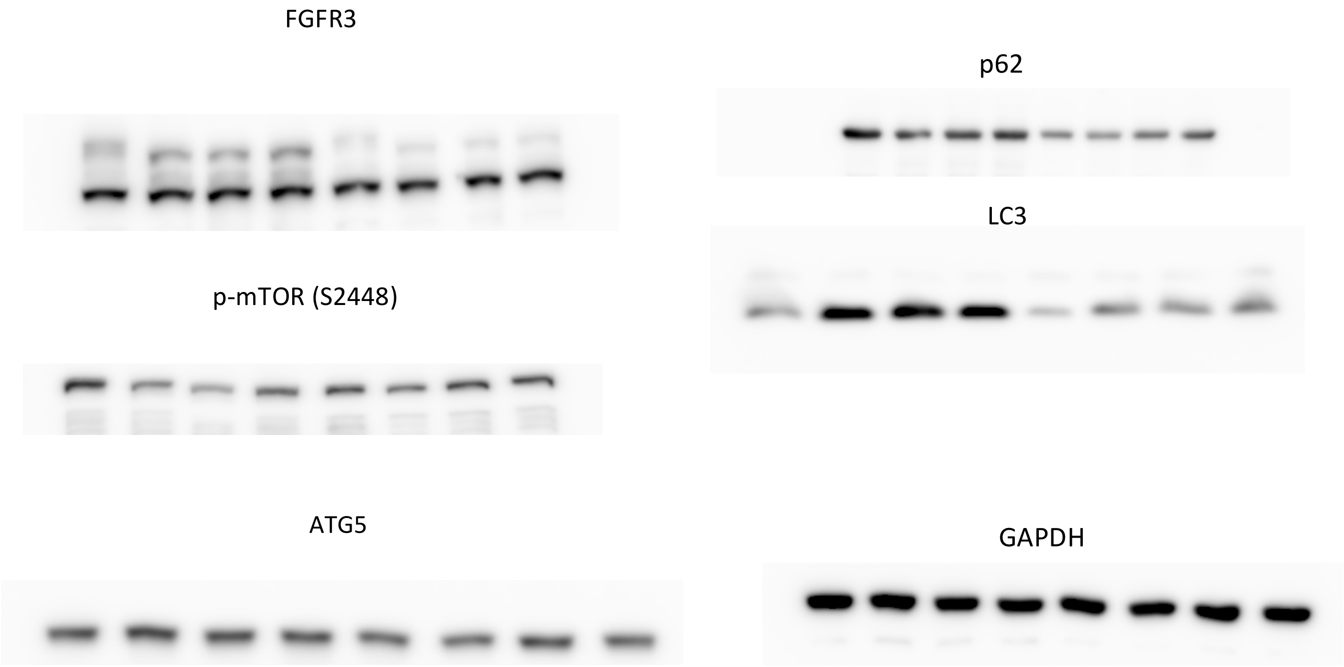

Figure 7C

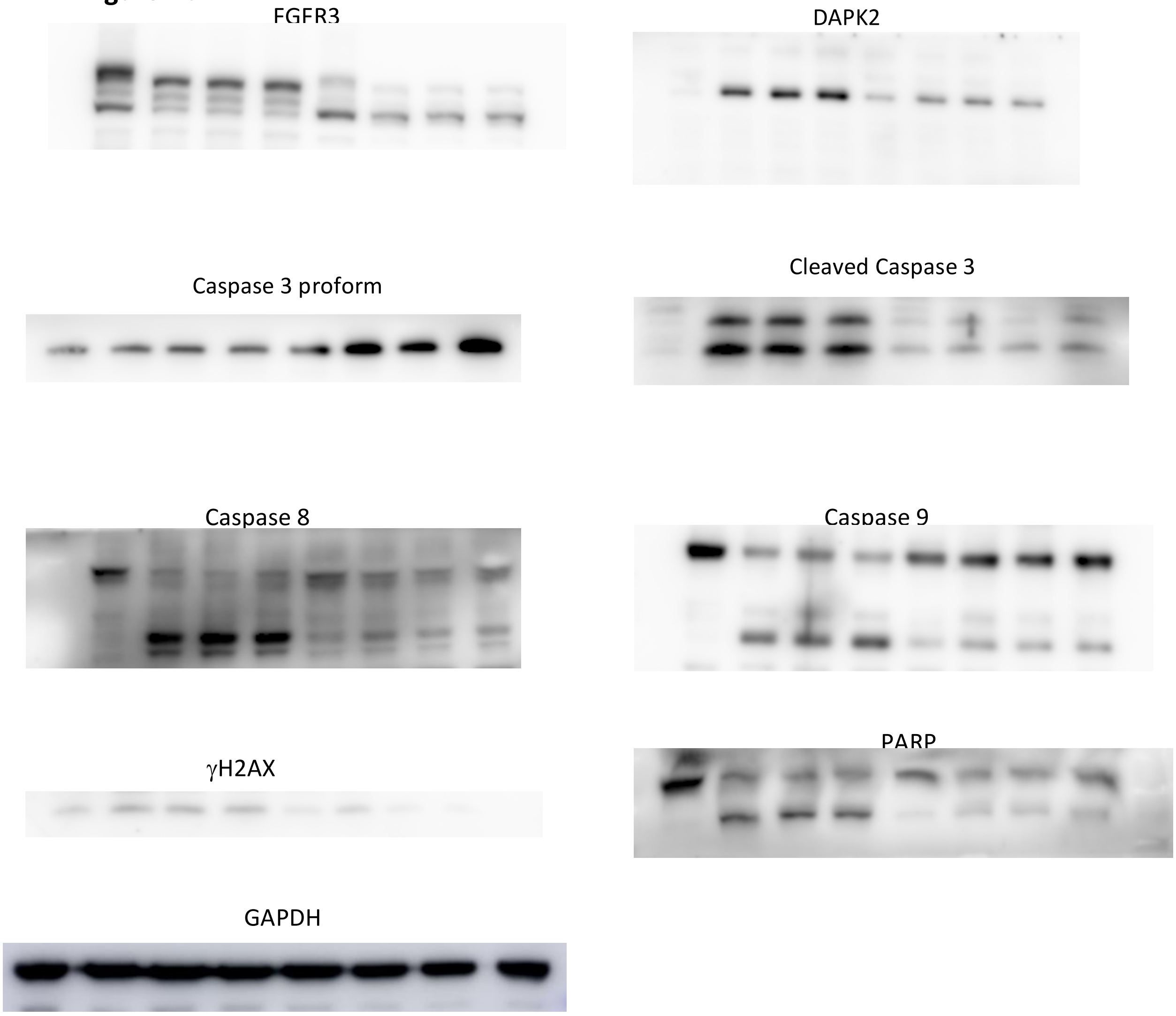
FGFR3
DAPK2
Caspase 3 proform
Cleaved Caspase 3
Caspase 8
Caspase 9
γH2AX
PARP
GAPDH

Figure S1A

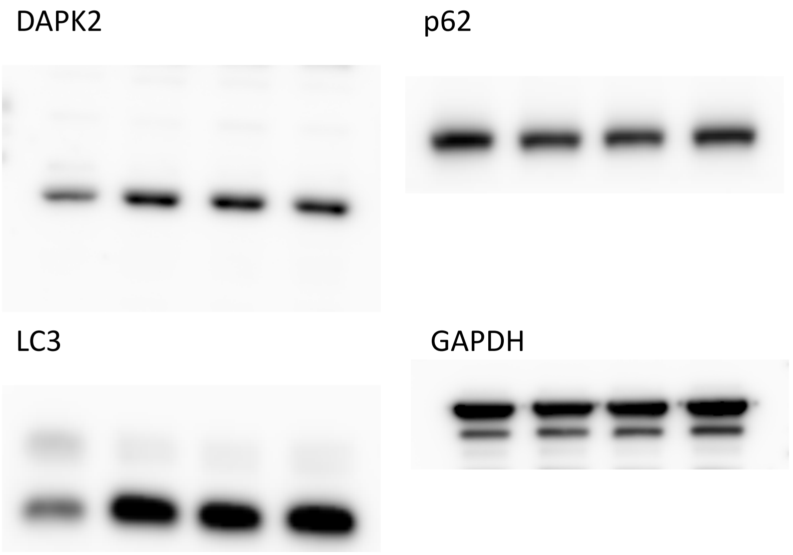

Figure S1B

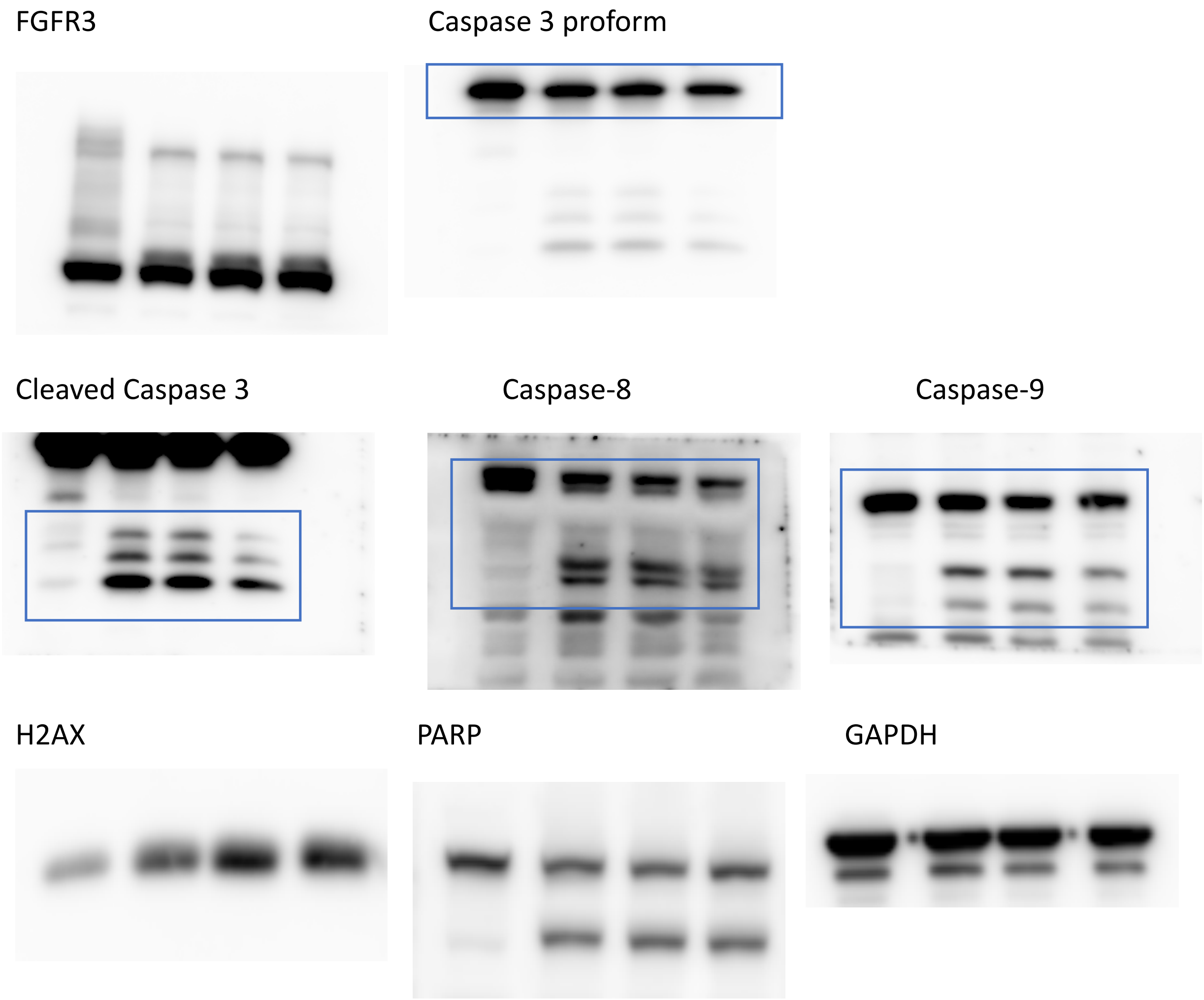

Supplement: Supplementary file 1 [file marinedrugs-21-00073-s001.zip › Original Images for Blots_Marine drugs_revision.pdf]
