# Supplementary material for: Stellettin B Induces Cell Death in Bladder Cancer Via Activating the Autophagy/DAPK2/Apoptosis Signaling Cascade
Source: Mar Drugs. 2023 Jan 21;21(2):73. doi: 10.3390/md21020073 (PMC9966069; doi:10.3390/md21020073)
Supplement: Supplementary file 1 [file marinedrugs-21-00073-s001.zip › Supplementary data for revision.pdf]

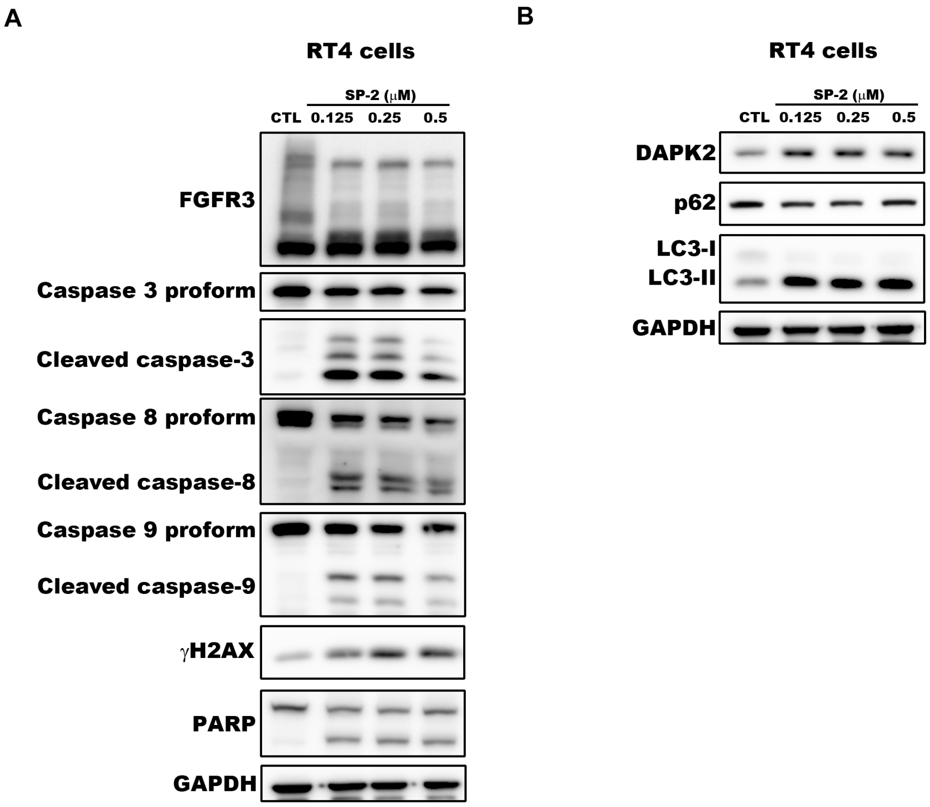

Figure S1. SP-2 induces autophagy and apoptosis in RT4 cells. (**A**). SP-2 causes band-shift of FGFR3-TACC3 fusion protein and induces apoptosis in RT4 cells. Cells were treated with different concentrations of SP-2 for 48 h, and subjected to western blotting using the indicated antibodies. (**B**) SP-2 induces DAPK2 expression and autophagy in RT4 cells. Cells were treated with different concentrations of SP-2 for 24 h, and subjected to western blotting using the indicated antibodies

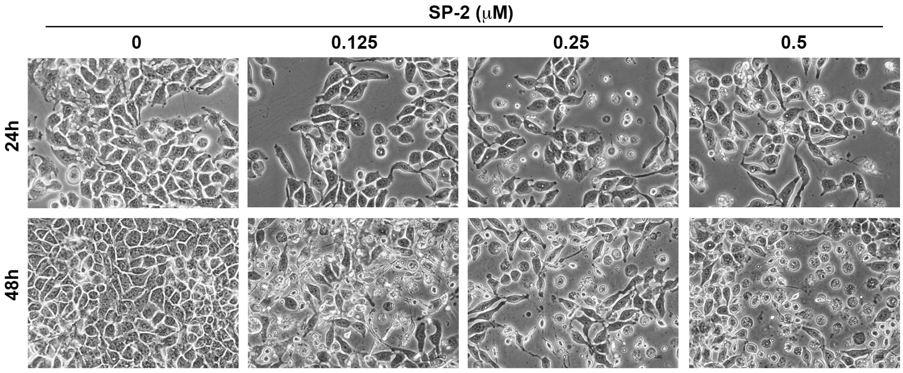

Figure S2. Morphological changes of RT-112 cells after the treatment of SP-2. RT-112 cells were treated with different concentrations of SP-2 for 24h and 48h, and the morphological changes were recorded by light microscopy. Scale bar = 1 μm.

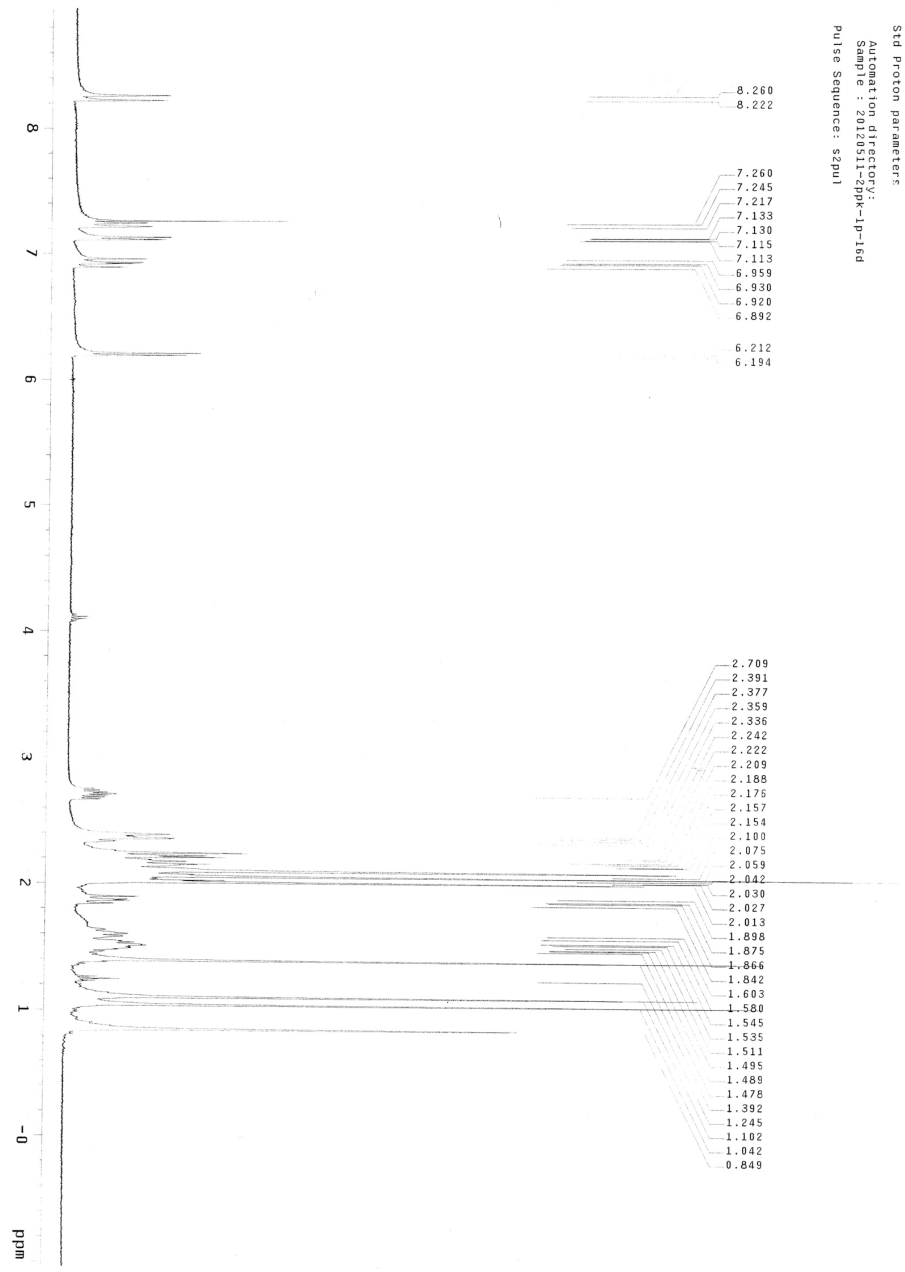

Figure S3. ^{1}H **NMR spectrum of SP-2 in** $CDCl_3$ **at 400 MHz.**

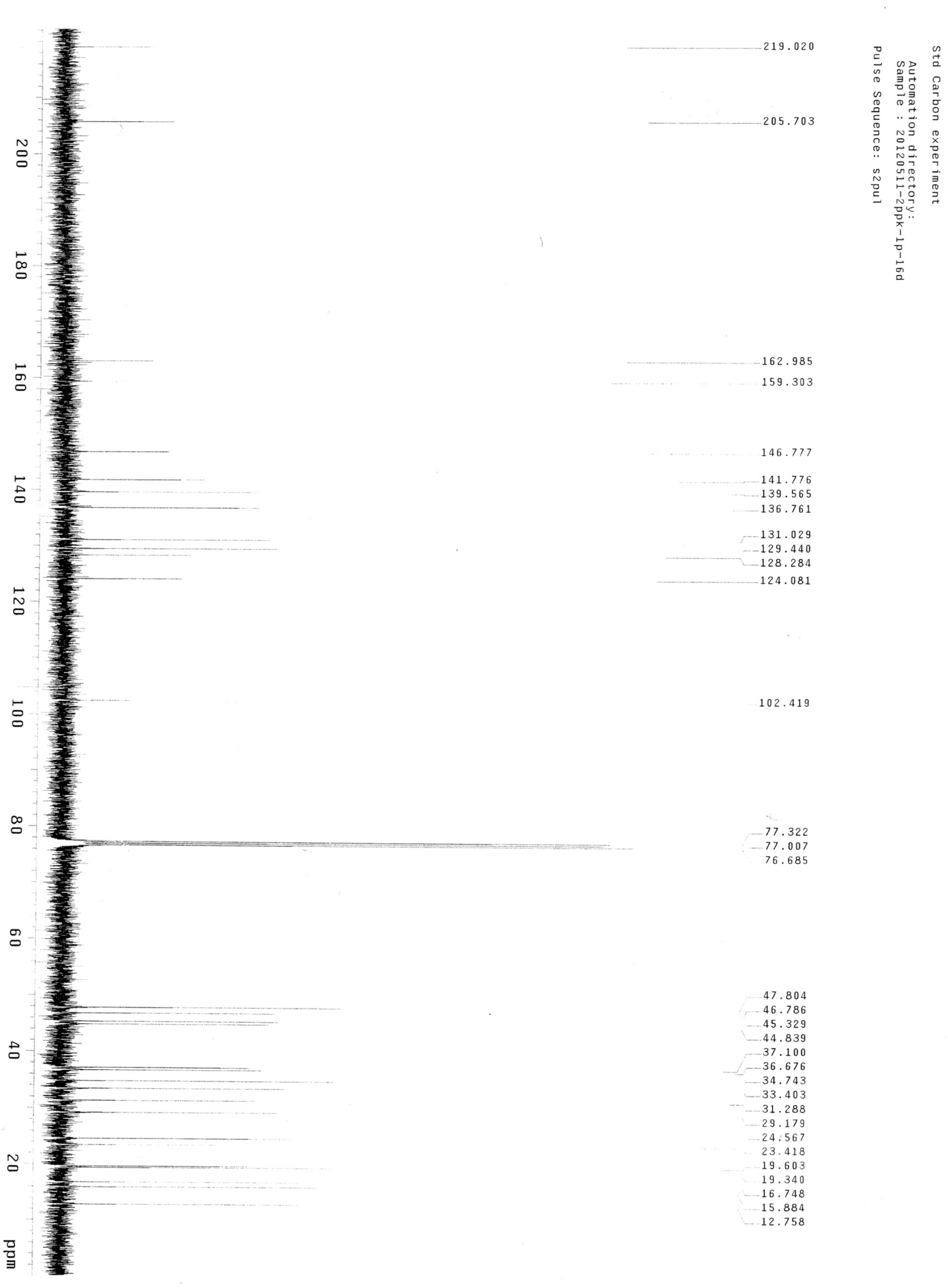

Figure S4. ^{13}C NMR spectrum of SP-2 in $CDCl_3$ at 100 MHz.
